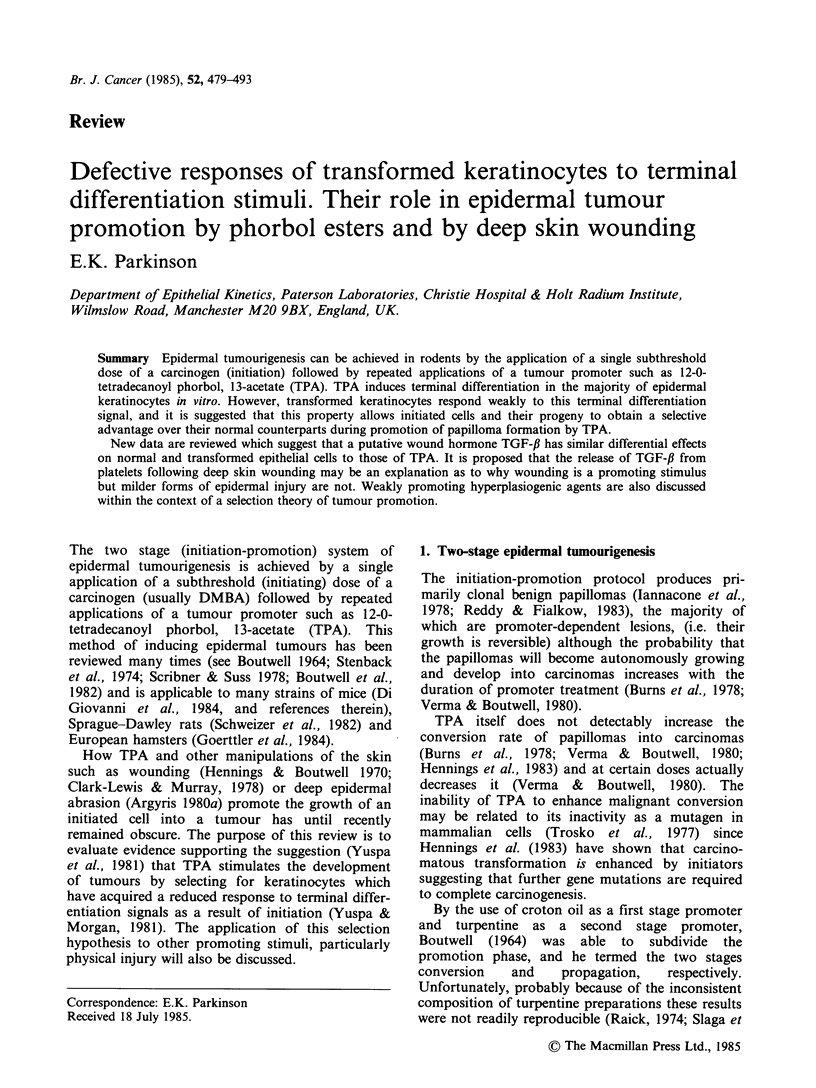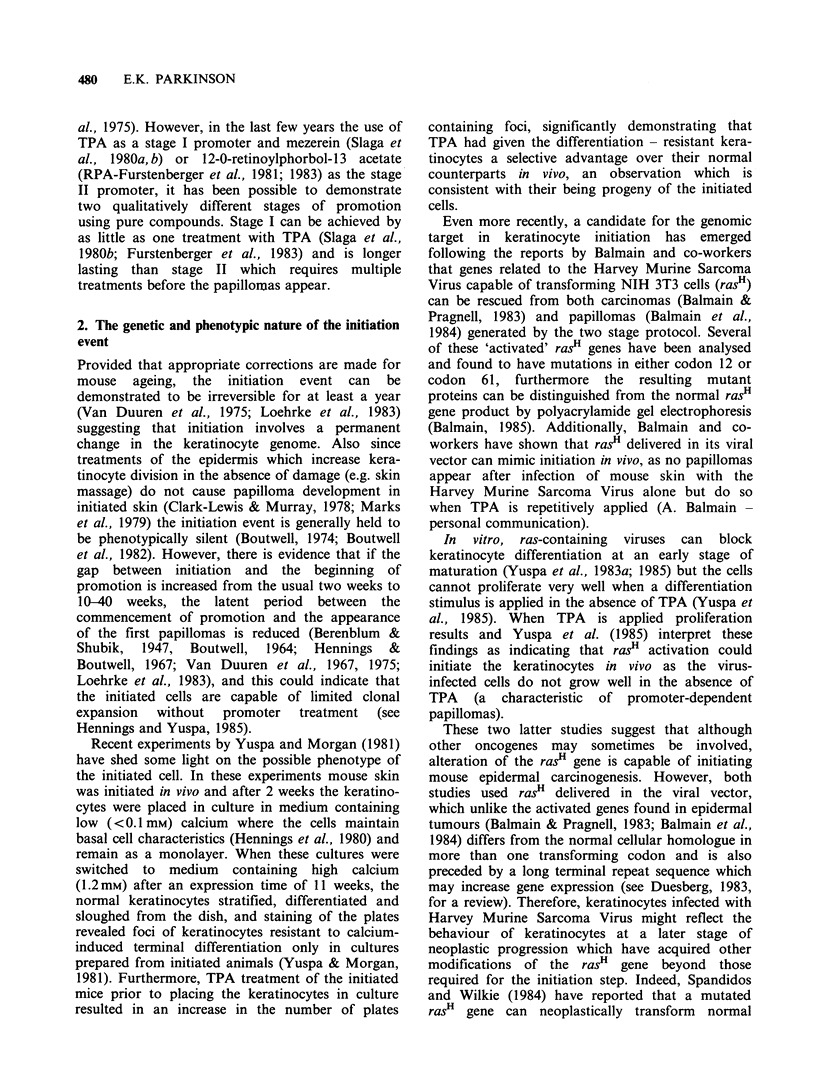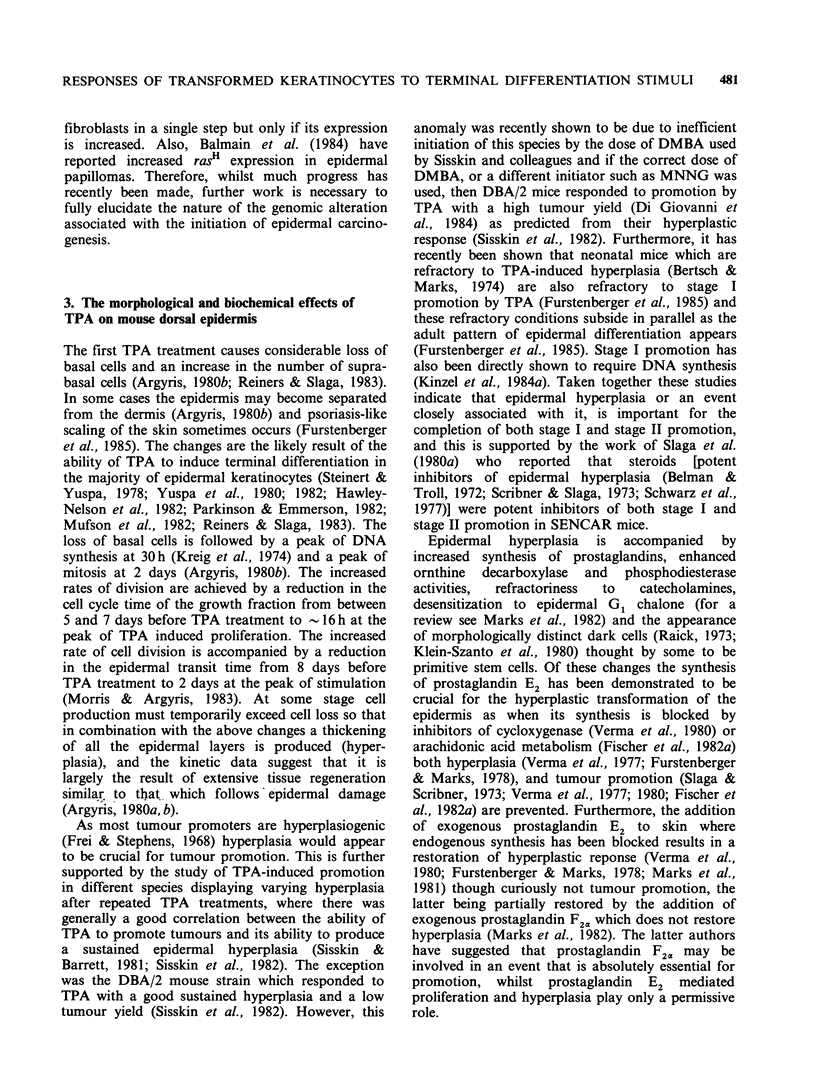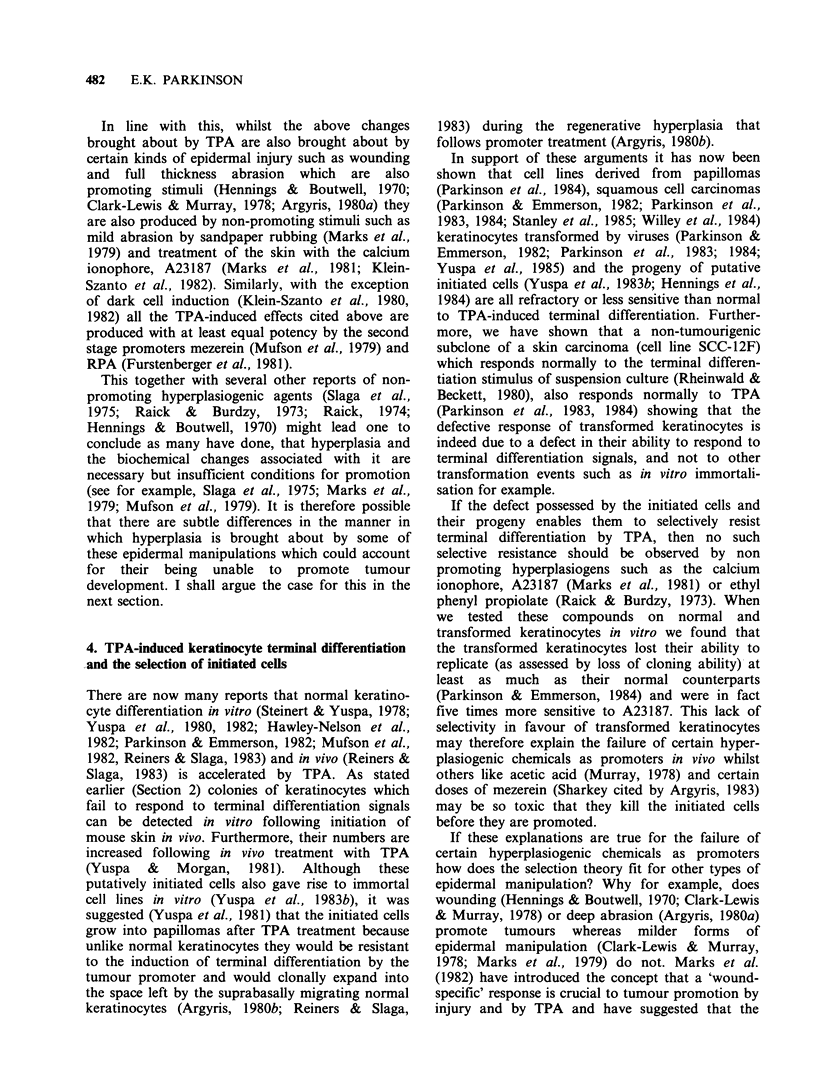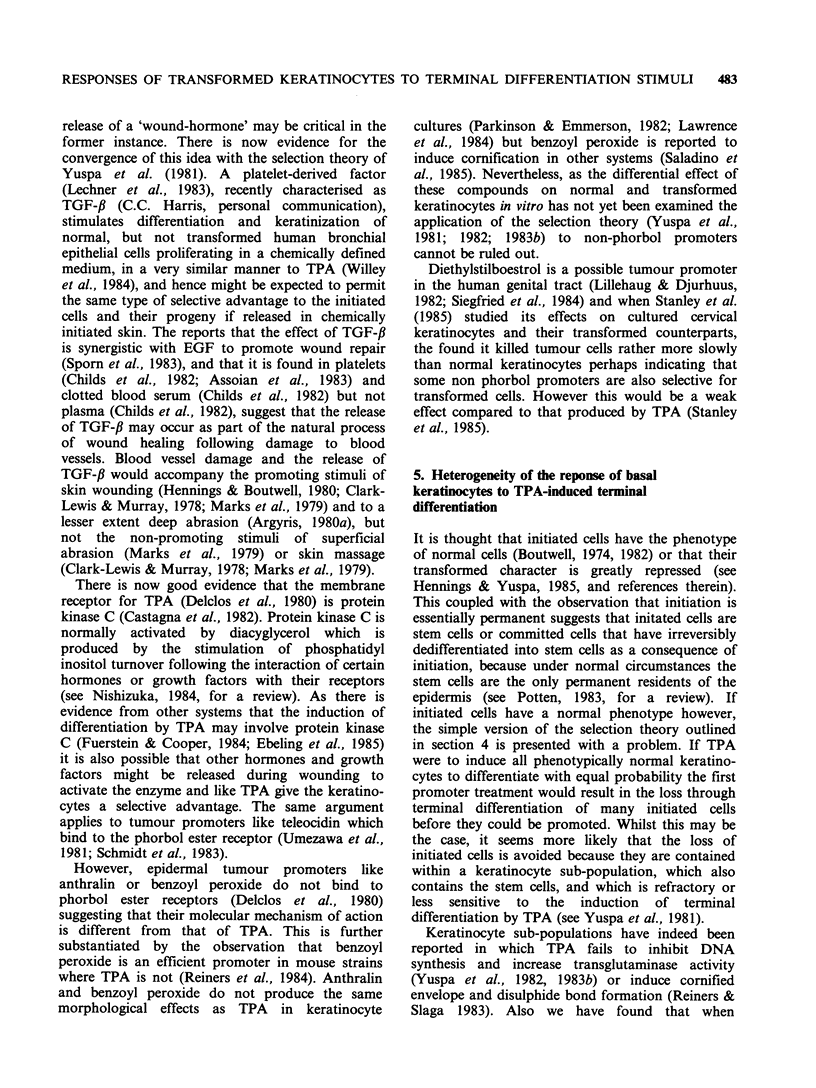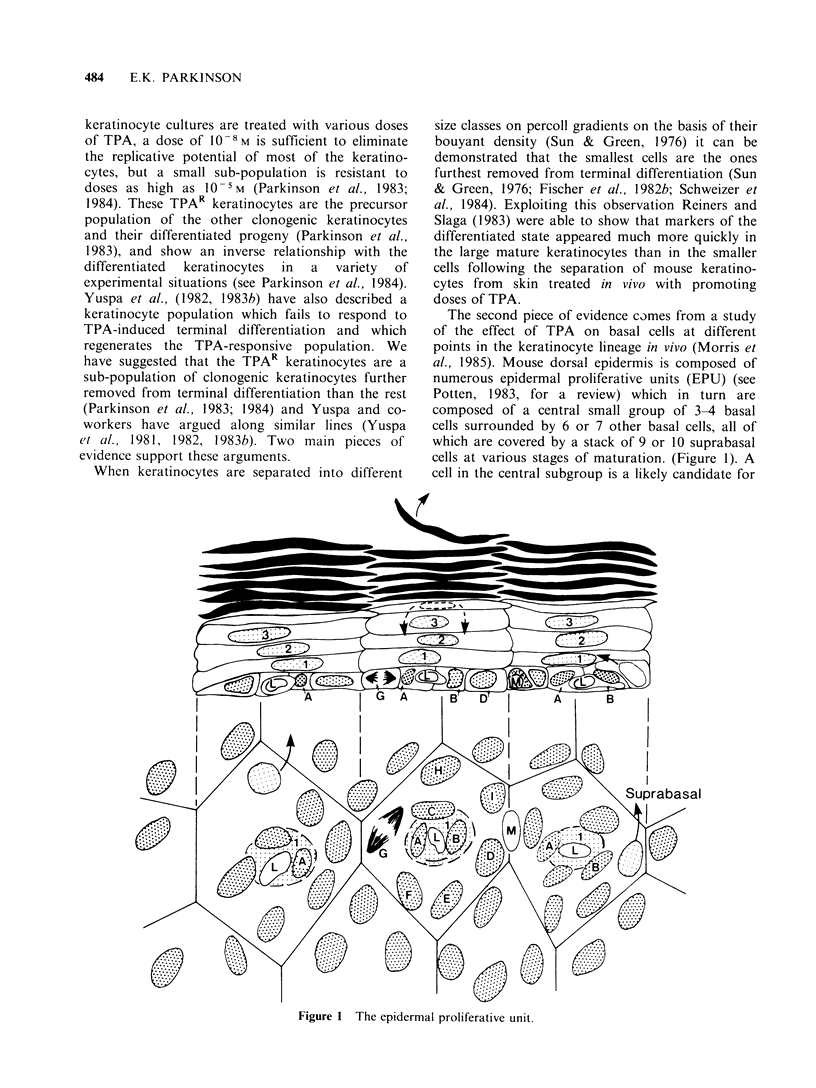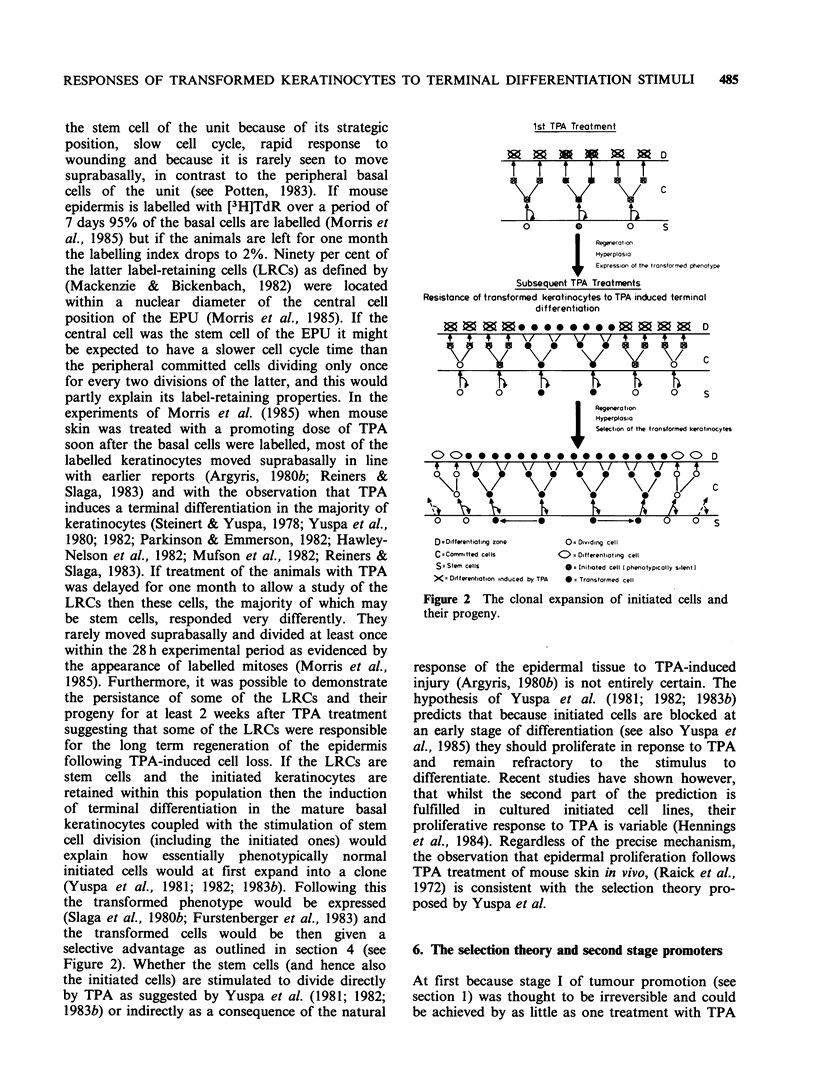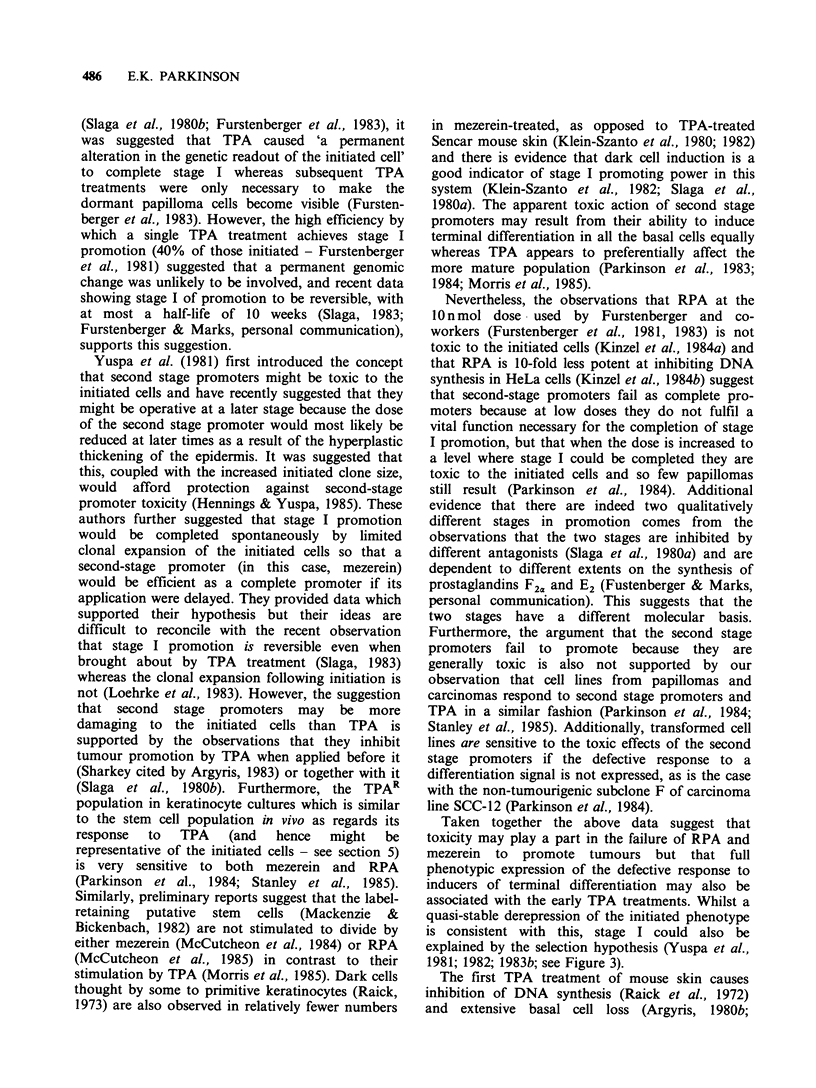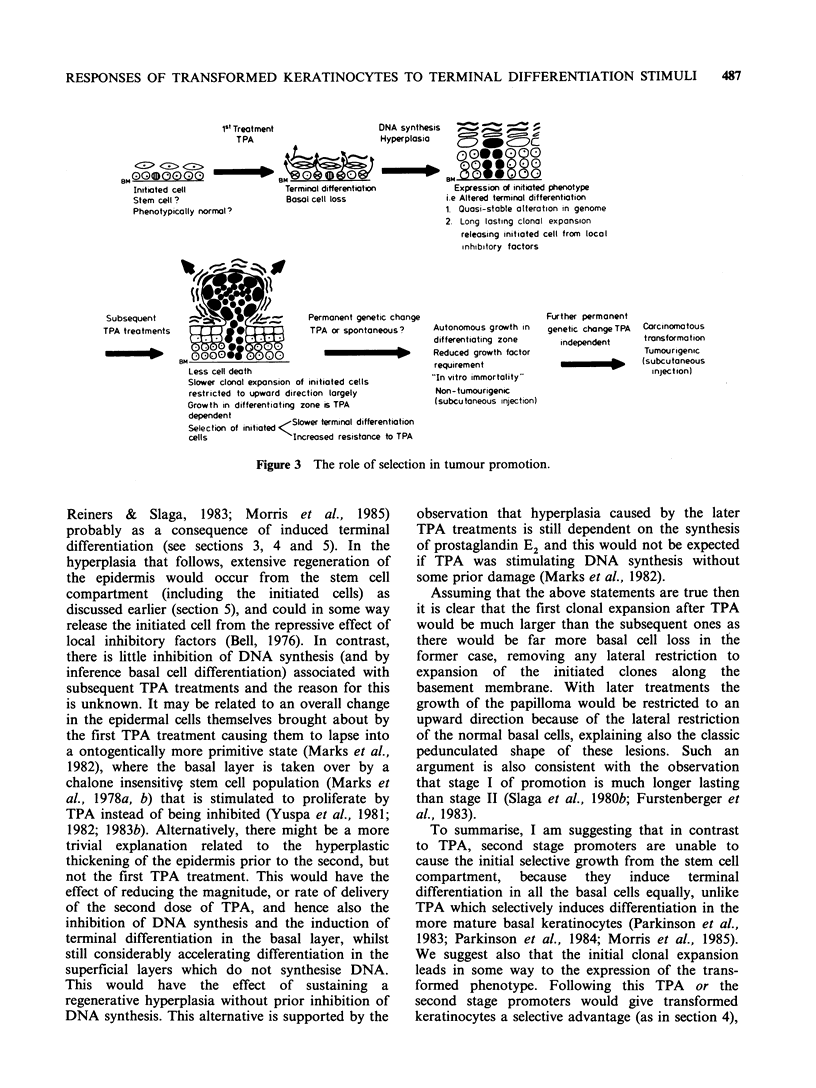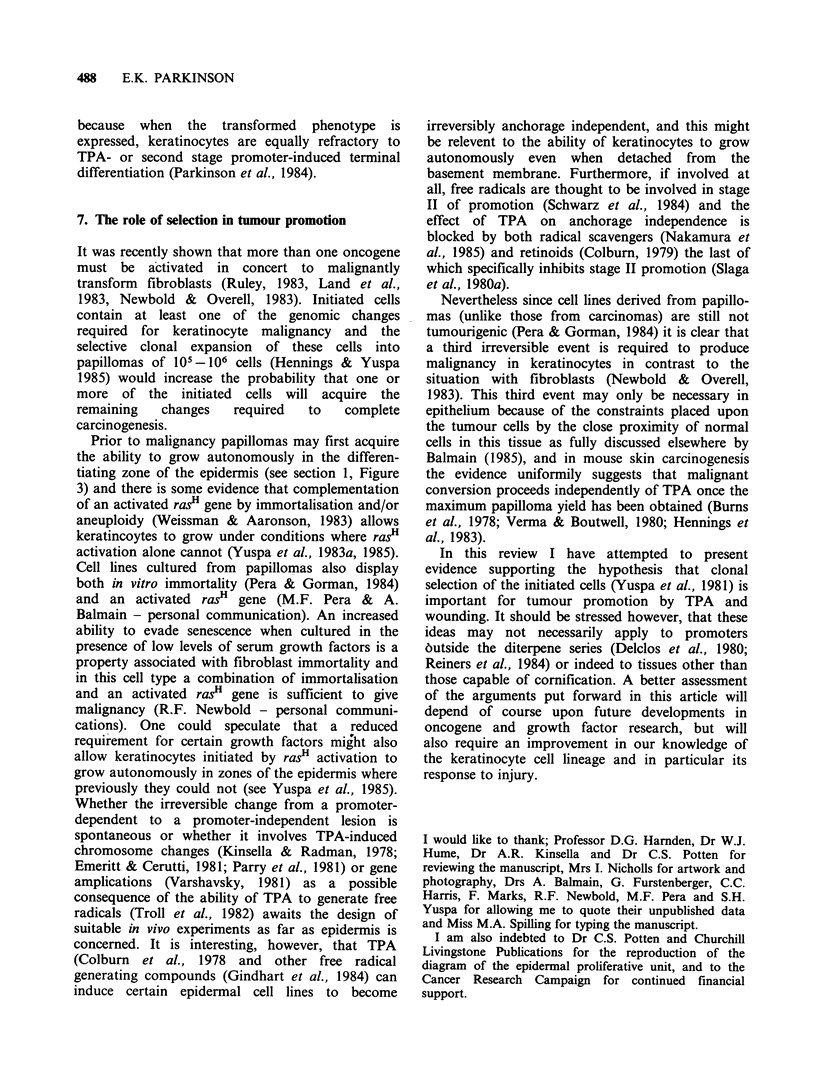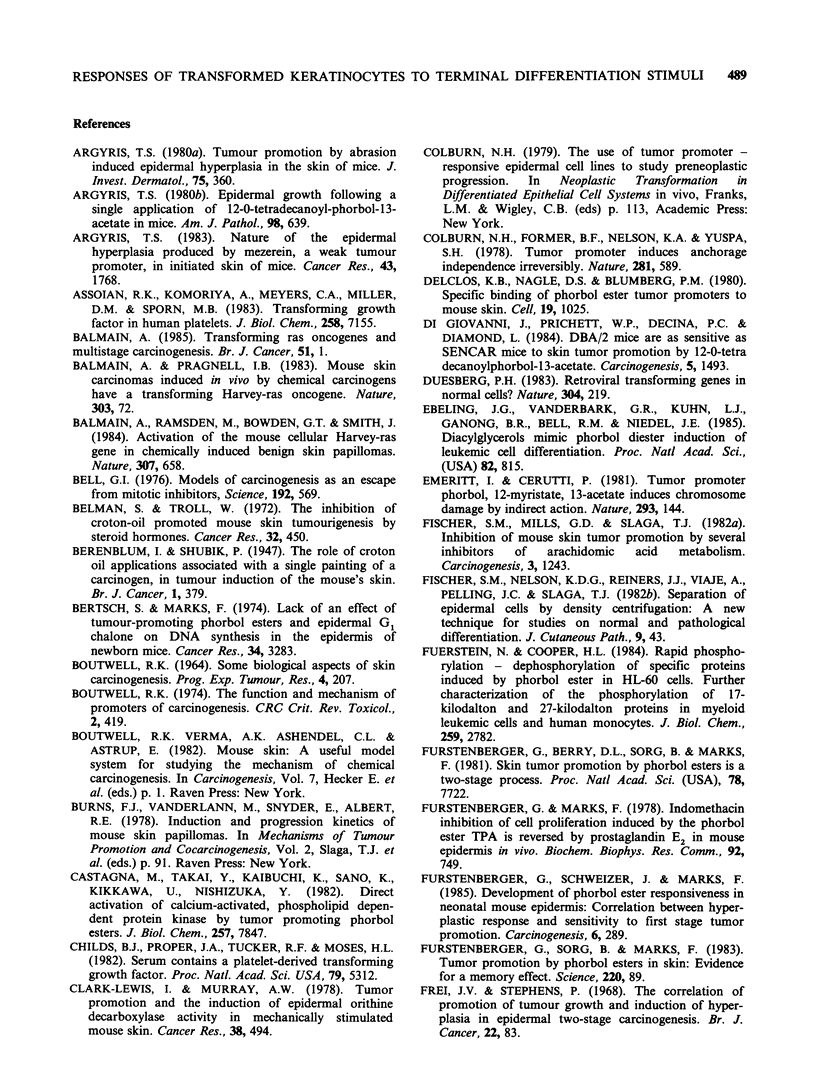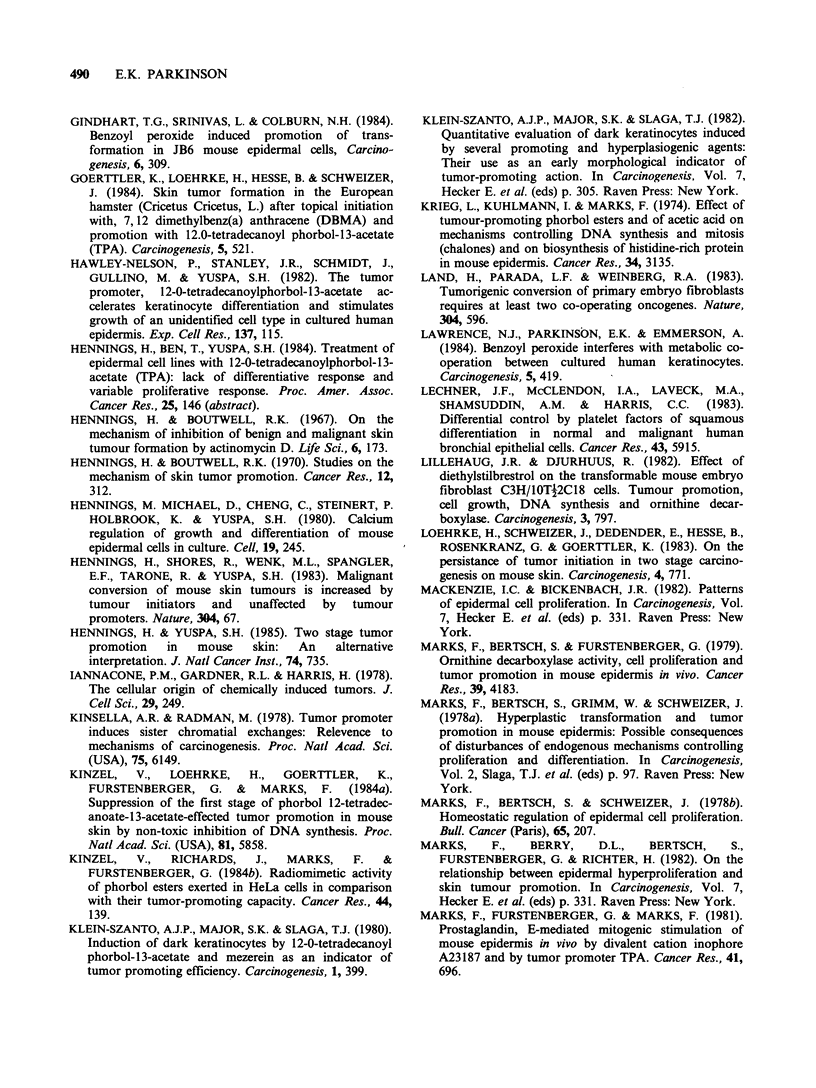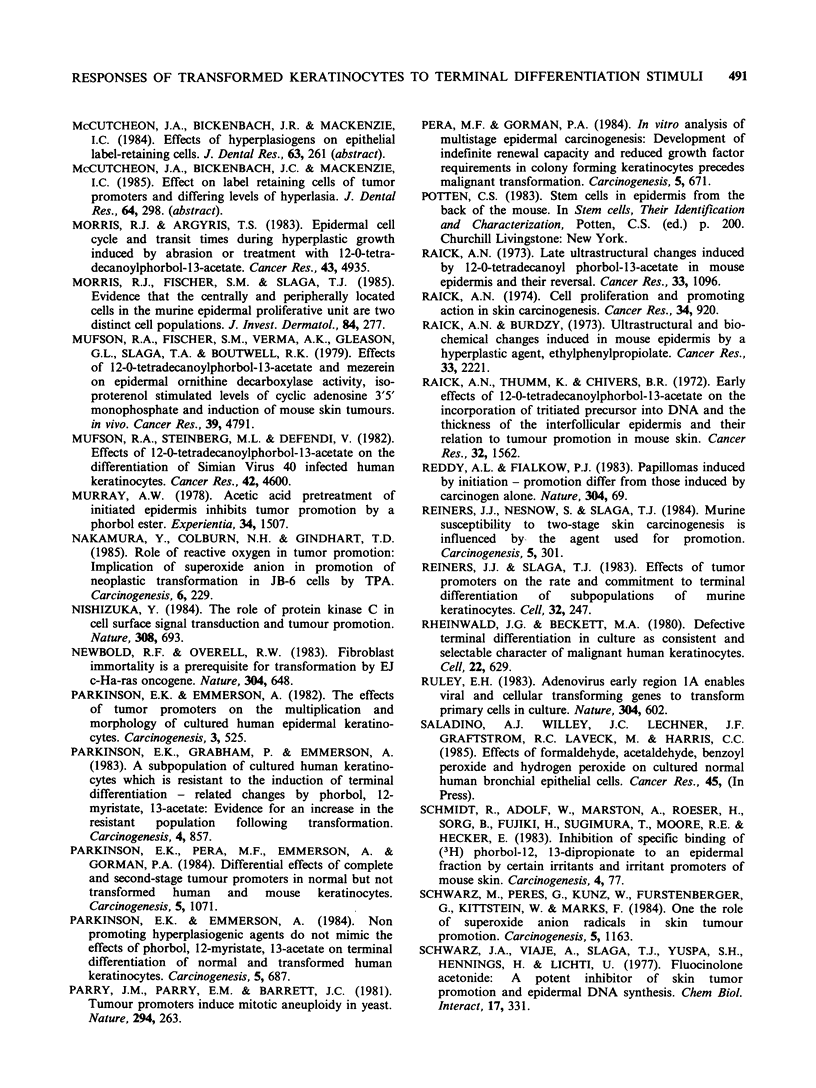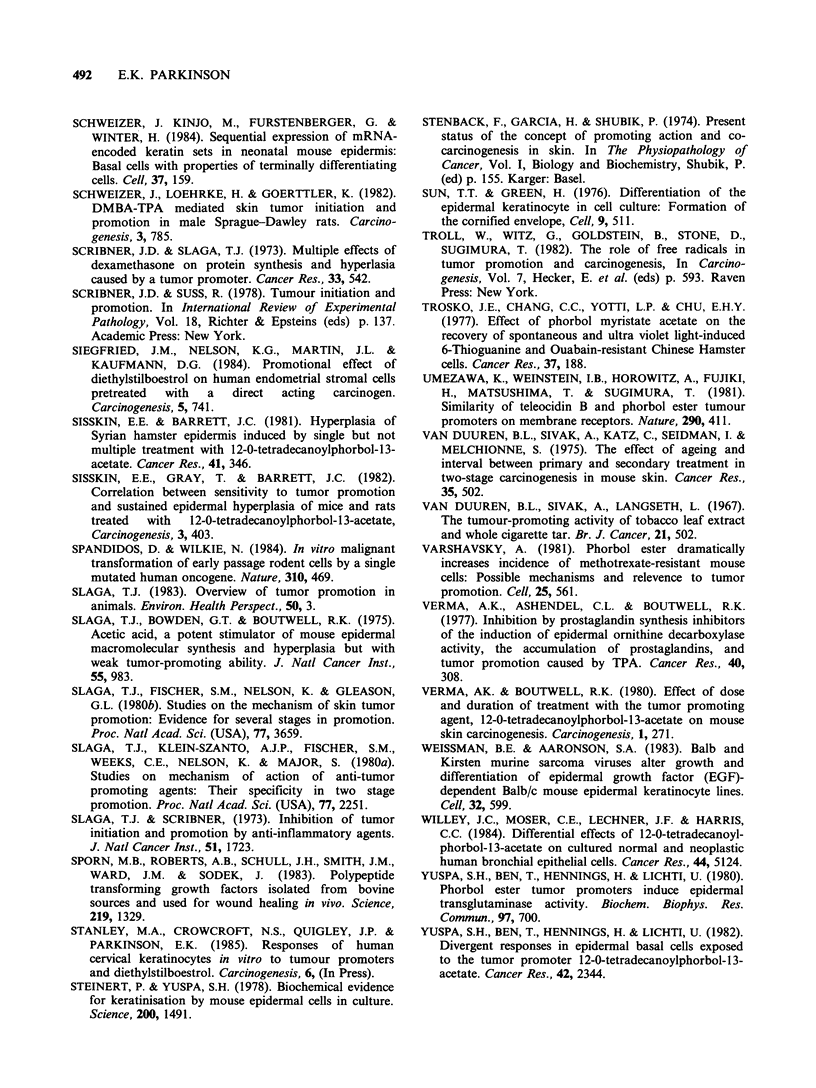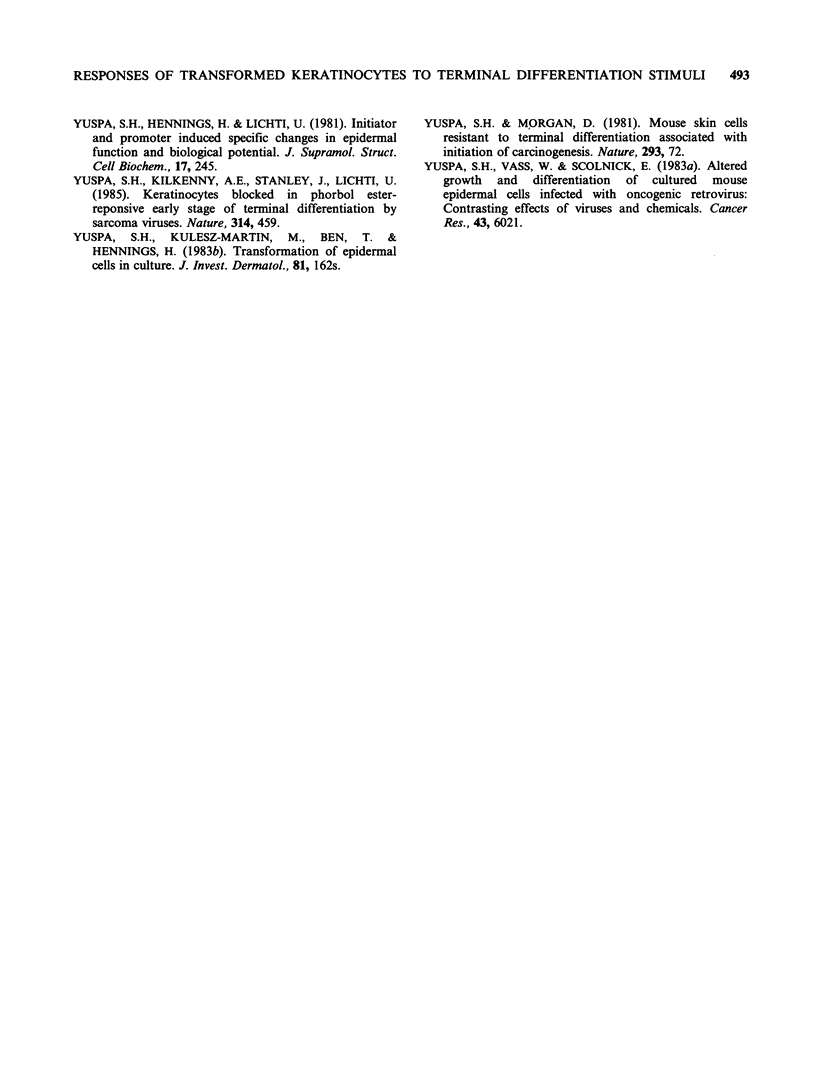# Defective responses of transformed keratinocytes to terminal differentiation stimuli. Their role in epidermal tumour promotion by phorbol esters and by deep skin wounding.

**DOI:** 10.1038/bjc.1985.219

**Published:** 1985-10

**Authors:** E. K. Parkinson

## Abstract

Epidermal tumourigenesis can be achieved in rodents by the application of a single subthreshold dose of a carcinogen (initiation) followed by repeated applications of a tumour promoter such as 12-0-tetradecanoyl phorbol, 13-acetate (TPA). TPA induces terminal differentiation in the majority of epidermal keratinocytes in vitro. However, transformed keratinocytes respond weakly to this terminal differentiation signal, and it is suggested that this property allows initiated cells and their progeny to obtain a selective advantage over their normal counterparts during promotion of papilloma formation by TPA. New data are reviewed which suggest that a putative wound hormone TGF-beta has similar differential effects on normal and transformed epithelial cells to those of TPA. It is proposed that the release of TGF-beta from platelets following deep skin wounding may be an explanation as to why wounding is a promoting stimulus but milder forms of epidermal injury are not. Weakly promoting hyperplasiogenic agents are also discussed within the context of a selection theory of tumour promotion.


*Br. J. Cancer* (1985), **52**, 479–493

**Review**

### Defective responses of transformed keratinocytes to terminal differentiation stimuli. Their role in epidermal tumour promotion by phorbol esters and by deep skin wounding

E.K. Parkinson

*Department of Epithelial Kinetics, Paterson Laboratories, Christie Hospital & Holt Radium Institute, Wilmslow Road, Manchester M20 9BX, England, UK.*

**Summary** Epidermal tumourigenesis can be achieved in rodents by the application of a single subthreshold dose of a carcinogen (initiation) followed by repeated applications of a tumour promoter such as 12-0-tetradecanoyl phorbol, 13-acetate (TPA). TPA induces terminal differentiation in the majority of epidermal keratinocytes *in vitro*. However, transformed keratinocytes respond weakly to this terminal differentiation signal, and it is suggested that this property allows initiated cells and their progeny to obtain a selective advantage over their normal counterparts during promotion of papilloma formation by TPA.

New data are reviewed which suggest that a putative wound hormone TGF-$\beta$ has similar differential effects on normal and transformed epithelial cells to those of TPA. It is proposed that the release of TGF-$\beta$ from platelets following deep skin wounding may be an explanation as to why wounding is a promoting stimulus but milder forms of epidermal injury are not. Weakly promoting hyperplasiogenic agents are also discussed within the context of a selection theory of tumour promotion.

The two stage (initiation-promotion) system of epidermal tumourigenesis is achieved by a single application of a subthreshold (initiating) dose of a carcinogen (usually DMBA) followed by repeated applications of a tumour promoter such as 12-0-tetradecanoyl phorbol, 13-acetate (TPA). This method of inducing epidermal tumours has been reviewed many times (see Boutwell 1964; Stenback *et al.*, 1974; Scribner & Suss 1978; Boutwell *et al.*, 1982) and is applicable to many strains of mice (Di Giovanni *et al.*, 1984, and references therein), Sprague–Dawley rats (Schweizer *et al.*, 1982) and European hamsters (Goerttler *et al.*, 1984).

How TPA and other manipulations of the skin such as wounding (Hennings & Boutwell 1970; Clark-Lewis & Murray, 1978) or deep epidermal abrasion (Argyris 1980*a*) promote the growth of an initiated cell into a tumour has until recently remained obscure. The purpose of this review is to evaluate evidence supporting the suggestion (Yuspa *et al.*, 1981) that TPA stimulates the development of tumours by selecting for keratinocytes which have acquired a reduced response to terminal differentiation signals as a result of initiation (Yuspa & Morgan, 1981). The application of this selection hypothesis to other promoting stimuli, particularly physical injury will also be discussed.

Correspondence: E.K. Parkinson
Received 18 July 1985.

### 1. Two-stage epidermal tumourigenesis

The initiation-promotion protocol produces primarily clonal benign papillomas (Iannacone *et al.*, 1978; Reddy & Fialkow, 1983), the majority of which are promoter-dependent lesions, (i.e. their growth is reversible) although the probability that the papillomas will become autonomously growing and develop into carcinomas increases with the duration of promoter treatment (Burns *et al.*, 1978; Verma & Boutwell, 1980).

TPA itself does not detectably increase the conversion rate of papillomas into carcinomas (Burns *et al.*, 1978; Verma & Boutwell, 1980; Hennings *et al.*, 1983) and at certain doses actually decreases it (Verma & Boutwell, 1980). The inability of TPA to enhance malignant conversion may be related to its inactivity as a mutagen in mammalian cells (Trosko *et al.*, 1977) since Hennings *et al.* (1983) have shown that carcinomatous transformation *is* enhanced by initiators suggesting that further gene mutations are required to complete carcinogenesis.

By the use of croton oil as a first stage promoter and turpentine as a second stage promoter, Boutwell (1964) was able to subdivide the promotion phase, and he termed the two stages conversion and propagation, respectively. Unfortunately, probably because of the inconsistent composition of turpentine preparations these results were not readily reproducible (Raick, 1974; Slaga *et*

© The Macmillan Press Ltd., 1985

*al.*, 1975). However, in the last few years the use of TPA as a stage I promoter and mezerein (Slaga *et al.*, 1980*a,b*) or 12-0-retinoylphorbol-13 acetate (RPA-Furstenberger *et al.*, 1981; 1983) as the stage II promoter, it has been possible to demonstrate two qualitatively different stages of promotion using pure compounds. Stage I can be achieved by as little as one treatment with TPA (Slaga *et al.*, 1980*b*; Furstenberger *et al.*, 1983) and is longer lasting than stage II which requires multiple treatments before the papillomas appear.

### 2. The genetic and phenotypic nature of the initiation event

Provided that appropriate corrections are made for mouse ageing, the initiation event can be demonstrated to be irreversible for at least a year (Van Duuren *et al.*, 1975; Loehrke *et al.*, 1983) suggesting that initiation involves a permanent change in the keratinocyte genome. Also since treatments of the epidermis which increase keratinocyte division in the absence of damage (e.g. skin massage) do not cause papilloma development in initiated skin (Clark-Lewis & Murray, 1978; Marks *et al.*, 1979) the initiation event is generally held to be phenotypically silent (Boutwell, 1974; Boutwell *et al.*, 1982). However, there is evidence that if the gap between initiation and the beginning of promotion is increased from the usual two weeks to 10–40 weeks, the latent period between the commencement of promotion and the appearance of the first papillomas is reduced (Berenblum & Shubik, 1947, Boutwell, 1964; Hennings & Boutwell, 1967; Van Duuren *et al.*, 1967, 1975; Loehrke *et al.*, 1983), and this could indicate that the initiated cells are capable of limited clonal expansion without promoter treatment (see Hennings and Yuspa, 1985).

Recent experiments by Yuspa and Morgan (1981) have shed some light on the possible phenotype of the initiated cell. In these experiments mouse skin was initiated *in vivo* and after 2 weeks the keratinocytes were placed in culture in medium containing low (<0.1 mM) calcium where the cells maintain basal cell characteristics (Hennings *et al.*, 1980) and remain as a monolayer. When these cultures were switched to medium containing high calcium (1.2 mM) after an expression time of 11 weeks, the normal keratinocytes stratified, differentiated and sloughed from the dish, and staining of the plates revealed foci of keratinocytes resistant to calcium-induced terminal differentiation only in cultures prepared from initiated animals (Yuspa & Morgan, 1981). Furthermore, TPA treatment of the initiated mice prior to placing the keratinocytes in culture resulted in an increase in the number of plates containing foci, significantly demonstrating that TPA had given the differentiation – resistant keratinocytes a selective advantage over their normal counterparts *in vivo*, an observation which is consistent with their being progeny of the initiated cells.

Even more recently, a candidate for the genomic target in keratinocyte initiation has emerged following the reports by Balmain and co-workers that genes related to the Harvey Murine Sarcoma Virus capable of transforming NIH 3T3 cells (*ras*$^{H}$) can be rescued from both carcinomas (Balmain & Pragnell, 1983) and papillomas (Balmain *et al.*, 1984) generated by the two stage protocol. Several of these 'activated' *ras*$^{H}$ genes have been analysed and found to have mutations in either codon 12 or codon 61, furthermore the resulting mutant proteins can be distinguished from the normal *ras*$^{H}$ gene product by polyacrylamide gel electrophoresis (Balmain, 1985). Additionally, Balmain and co-workers have shown that *ras*$^{H}$ delivered in its viral vector can mimic initiation *in vivo*, as no papillomas appear after infection of mouse skin with the Harvey Murine Sarcoma Virus alone but do so when TPA is repetitively applied (A. Balmain – personal communication).

*In vitro, ras*-containing viruses can block keratinocyte differentiation at an early stage of maturation (Yuspa *et al.*, 1983*a*; 1985) but the cells cannot proliferate very well when a differentiation stimulus is applied in the absence of TPA (Yuspa *et al.*, 1985). When TPA is applied proliferation results and Yuspa *et al.* (1985) interpret these findings as indicating that *ras*$^{H}$ activation could initiate the keratinocytes *in vivo* as the virus-infected cells do not grow well in the absence of TPA (a characteristic of promoter-dependent papillomas).

These two latter studies suggest that although other oncogenes may sometimes be involved, alteration of the *ras*$^{H}$ gene is capable of initiating mouse epidermal carcinogenesis. However, both studies used *ras*$^{H}$ delivered in the viral vector, which unlike the activated genes found in epidermal tumours (Balmain & Pragnell, 1983; Balmain *et al.*, 1984) differs from the normal cellular homologue in more than one transforming codon and is also preceded by a long terminal repeat sequence which may increase gene expression (see Duesberg, 1983, for a review). Therefore, keratinocytes infected with Harvey Murine Sarcoma Virus might reflect the behaviour of keratinocytes at a later stage of neoplastic progression which have acquired other modifications of the *ras*$^{H}$ gene beyond those required for the initiation step. Indeed, Spandidos and Wilkie (1984) have reported that a mutated *ras*$^{H}$ gene can neoplastically transform normal fibroblasts in a single step but only if its expression is increased. Also, Balmain *et al.* (1984) have reported increased *ras*$^{H}$ expression in epidermal papillomas. Therefore, whilst much progress has recently been made, further work is necessary to fully elucidate the nature of the genomic alteration associated with the initiation of epidermal carcinogenesis.

### 3. The morphological and biochemical effects of TPA on mouse dorsal epidermis

The first TPA treatment causes considerable loss of basal cells and an increase in the number of suprabasal cells (Argyris, 1980*b*; Reiners & Slaga, 1983). In some cases the epidermis may become separated from the dermis (Argyris, 1980*b*) and psoriasis-like scaling of the skin sometimes occurs (Furstenberger *et al.*, 1985). The changes are the likely result of the ability of TPA to induce terminal differentiation in the majority of epidermal keratinocytes (Steinert & Yuspa, 1978; Yuspa *et al.*, 1980; 1982; Hawley-Nelson *et al.*, 1982; Parkinson & Emmerson, 1982; Mufson *et al.*, 1982; Reiners & Slaga, 1983). The loss of basal cells is followed by a peak of DNA synthesis at 30 h (Kreig *et al.*, 1974) and a peak of mitosis at 2 days (Argyris, 1980*b*). The increased rates of division are achieved by a reduction in the cell cycle time of the growth fraction from between 5 and 7 days before TPA treatment to ~16 h at the peak of TPA induced proliferation. The increased rate of cell division is accompanied by a reduction in the epidermal transit time from 8 days before TPA treatment to 2 days at the peak of stimulation (Morris & Argyris, 1983). At some stage cell production must temporarily exceed cell loss so that in combination with the above changes a thickening of all the epidermal layers is produced (hyperplasia), and the kinetic data suggest that it is largely the result of extensive tissue regeneration similar to that which follows epidermal damage (Argyris, 1980*a, b*).

As most tumour promoters are hyperplasiogenic (Frei & Stephens, 1968) hyperplasia would appear to be crucial for tumour promotion. This is further supported by the study of TPA-induced promotion in different species displaying varying hyperplasia after repeated TPA treatments, where there was generally a good correlation between the ability of TPA to promote tumours and its ability to produce a sustained epidermal hyperplasia (Sisskin & Barrett, 1981; Sisskin *et al.*, 1982). The exception was the DBA/2 mouse strain which responded to TPA with a good sustained hyperplasia and a low tumour yield (Sisskin *et al.*, 1982). However, this anomaly was recently shown to be due to inefficient initiation of this species by the dose of DMBA used by Sisskin and colleagues and if the correct dose of DMBA, or a different initiator such as MNNG was used, then DBA/2 mice responded to promotion by TPA with a high tumour yield (Di Giovanni *et al.*, 1984) as predicted from their hyperplastic response (Sisskin *et al.*, 1982). Furthermore, it has recently been shown that neonatal mice which are refractory to TPA-induced hyperplasia (Bertsch & Marks, 1974) are also refractory to stage I promotion by TPA (Furstenberger *et al.*, 1985) and these refractory conditions subside in parallel as the adult pattern of epidermal differentiation appears (Furstenberger *et al.*, 1985). Stage I promotion has also been directly shown to require DNA synthesis (Kinzel *et al.*, 1984*a*). Taken together these studies indicate that epidermal hyperplasia or an event closely associated with it, is important for the completion of both stage I and stage II promotion, and this is supported by the work of Slaga *et al.* (1980*a*) who reported that steroids [potent inhibitors of epidermal hyperplasia (Belman & Troll, 1972; Scribner & Slaga, 1973; Schwarz *et al.*, 1977)] were potent inhibitors of both stage I and stage II promotion in SENCAR mice.

Epidermal hyperplasia is accompanied by increased synthesis of prostaglandins, enhanced ornthine decarboxylase and phosphodiesterase activities, refractoriness to catecholamines, desensitization to epidermal $G_1$ chalone (for a review see Marks *et al.*, 1982) and the appearance of morphologically distinct dark cells (Raick, 1973; Klein-Szanto *et al.*, 1980) thought by some to be primitive stem cells. Of these changes the synthesis of prostaglandin $E_2$ has been demonstrated to be crucial for the hyperplastic transformation of the epidermis as when its synthesis is blocked by inhibitors of cycloxygenase (Verma *et al.*, 1980) or arachidonic acid metabolism (Fischer *et al.*, 1982*a*) both hyperplasia (Verma *et al.*, 1977; Furstenberger & Marks, 1978), and tumour promotion (Slaga & Scribner, 1973; Verma *et al.*, 1977; 1980; Fischer *et al.*, 1982*a*) are prevented. Furthermore, the addition of exogenous prostaglandin $E_2$ to skin where endogenous synthesis has been blocked results in a restoration of hyperplastic reponse (Verma *et al.*, 1980; Furstenberger & Marks, 1978; Marks *et al.*, 1981) though curiously not tumour promotion, the latter being partially restored by the addition of exogenous prostaglandin $F_{2\alpha}$ which does not restore hyperplasia (Marks *et al.*, 1982). The latter authors have suggested that prostaglandin $F_{2\alpha}$ may be involved in an event that is absolutely essential for promotion, whilst prostaglandin $E_2$ mediated proliferation and hyperplasia play only a permissive role.

In line with this, whilst the above changes brought about by TPA are also brought about by certain kinds of epidermal injury such as wounding and full thickness abrasion which are also promoting stimuli (Hennings & Boutwell, 1970; Clark-Lewis & Murray, 1978; Argyris, 1980*a*) they are also produced by non-promoting stimuli such as mild abrasion by sandpaper rubbing (Marks *et al.*, 1979) and treatment of the skin with the calcium ionophore, A23187 (Marks *et al.*, 1981; Klein-Szanto *et al.*, 1982). Similarly, with the exception of dark cell induction (Klein-Szanto *et al.*, 1980, 1982) all the TPA-induced effects cited above are produced with at least equal potency by the second stage promoters mezerein (Mufson *et al.*, 1979) and RPA (Furstenberger *et al.*, 1981).

This together with several other reports of non-promoting hyperplasiogenic agents (Slaga *et al.*, 1975; Raick & Burdzy, 1973; Raick, 1974; Hennings & Boutwell, 1970) might lead one to conclude as many have done, that hyperplasia and the biochemical changes associated with it are necessary but insufficient conditions for promotion (see for example, Slaga *et al.*, 1975; Marks *et al.*, 1979; Mufson *et al.*, 1979). It is therefore possible that there are subtle differences in the manner in which hyperplasia is brought about by some of these epidermal manipulations which could account for their being unable to promote tumour development. I shall argue the case for this in the next section.

### 4. TPA-induced keratinocyte terminal differentiation and the selection of initiated cells

There are now many reports that normal keratinocyte differentiation *in vitro* (Steinert & Yuspa, 1978; Yuspa *et al.*, 1980, 1982; Hawley-Nelson *et al.*, 1982; Parkinson & Emmerson, 1982; Mufson *et al.*, 1982, Reiners & Slaga, 1983) and *in vivo* (Reiners & Slaga, 1983) is accelerated by TPA. As stated earlier (Section 2) colonies of keratinocytes which fail to respond to terminal differentiation signals can be detected *in vitro* following initiation of mouse skin *in vivo*. Furthermore, their numbers are increased following *in vivo* treatment with TPA (Yuspa & Morgan, 1981). Although these putatively initiated cells also gave rise to immortal cell lines *in vitro* (Yuspa *et al.*, 1983*b*), it was suggested (Yuspa *et al.*, 1981) that the initiated cells grow into papillomas after TPA treatment because unlike normal keratinocytes they would be resistant to the induction of terminal differentiation by the tumour promoter and would clonally expand into the space left by the suprabasally migrating normal keratinocytes (Argyris, 1980*b*; Reiners & Slaga, 1983) during the regenerative hyperplasia that follows promoter treatment (Argyris, 1980*b*).

In support of these arguments it has now been shown that cell lines derived from papillomas (Parkinson *et al.*, 1984), squamous cell carcinomas (Parkinson & Emmerson, 1982; Parkinson *et al.*, 1983, 1984; Stanley *et al.*, 1985; Willey *et al.*, 1984) keratinocytes transformed by viruses (Parkinson & Emmerson, 1982; Parkinson *et al.*, 1983; 1984; Yuspa *et al.*, 1985) and the progeny of putative initiated cells (Yuspa *et al.*, 1983*b*; Hennings *et al.*, 1984) are all refractory or less sensitive than normal to TPA-induced terminal differentiation. Furthermore, we have shown that a non-tumourigenic subclone of a skin carcinoma (cell line SCC-12F) which responds normally to the terminal differentiation stimulus of suspension culture (Rheinwald & Beckett, 1980), also responds normally to TPA (Parkinson *et al.*, 1983, 1984) showing that the defective response of transformed keratinocytes is indeed due to a defect in their ability to respond to terminal differentiation signals, and not to other transformation events such as *in vitro* immortalisation for example.

If the defect possessed by the initiated cells and their progeny enables them to selectively resist terminal differentiation by TPA, then no such selective resistance should be observed by non promoting hyperplasiogens such as the calcium ionophore, A23187 (Marks *et al.*, 1981) or ethyl phenyl propiolate (Raick & Burdzy, 1973). When we tested these compounds on normal and transformed keratinocytes *in vitro* we found that the transformed keratinocytes lost their ability to replicate (as assessed by loss of cloning ability) at least as much as their normal counterparts (Parkinson & Emmerson, 1984) and were in fact five times more sensitive to A23187. This lack of selectivity in favour of transformed keratinocytes may therefore explain the failure of certain hyperplasiogenic chemicals as promoters *in vivo* whilst others like acetic acid (Murray, 1978) and certain doses of mezerein (Sharkey cited by Argyris, 1983) may be so toxic that they kill the initiated cells before they are promoted.

If these explanations are true for the failure of certain hyperplasiogenic chemicals as promoters how does the selection theory fit for other types of epidermal manipulation? Why for example, does wounding (Hennings & Boutwell, 1970; Clark-Lewis & Murray, 1978) or deep abrasion (Argyris, 1980*a*) promote tumours whereas milder forms of epidermal manipulation (Clark-Lewis & Murray, 1978; Marks *et al.*, 1979) do not. Marks *et al.* (1982) have introduced the concept that a 'wound-specific' response is crucial to tumour promotion by injury and by TPA and have suggested that the release of a 'wound-hormone' may be critical in the former instance. There is now evidence for the convergence of this idea with the selection theory of Yuspa *et al.* (1981). A platelet-derived factor (Lechner *et al.*, 1983), recently characterised as TGF-$\beta$ (C.C. Harris, personal communication), stimulates differentiation and keratinization of normal, but not transformed human bronchial epithelial cells proliferating in a chemically defined medium, in a very similar manner to TPA (Willey *et al.*, 1984), and hence might be expected to permit the same type of selective advantage to the initiated cells and their progeny if released in chemically initiated skin. The reports that the effect of TGF-$\beta$ is synergistic with EGF to promote wound repair (Sporn *et al.*, 1983), and that it is found in platelets (Childs *et al.*, 1982; Assoian *et al.*, 1983) and clotted blood serum (Childs *et al.*, 1982) but not plasma (Childs *et al.*, 1982), suggest that the release of TGF-$\beta$ may occur as part of the natural process of wound healing following damage to blood vessels. Blood vessel damage and the release of TGF-$\beta$ would accompany the promoting stimuli of skin wounding (Hennings & Boutwell, 1980; Clark-Lewis & Murray, 1978; Marks *et al.*, 1979) and to a lesser extent deep abrasion (Argyris, 1980*a*), but not the non-promoting stimuli of superficial abrasion (Marks *et al.*, 1979) or skin massage (Clark-Lewis & Murray, 1978; Marks *et al.*, 1979).

There is now good evidence that the membrane receptor for TPA (Delclos *et al.*, 1980) is protein kinase C (Castagna *et al.*, 1982). Protein kinase C is normally activated by diacyglycerol which is produced by the stimulation of phosphatidyl inositol turnover following the interaction of certain hormones or growth factors with their receptors (see Nishizuka, 1984, for a review). As there is evidence from other systems that the induction of differentiation by TPA may involve protein kinase C (Fuerstein & Cooper, 1984; Ebeling *et al.*, 1985) it is also possible that other hormones and growth factors might be released during wounding to activate the enzyme and like TPA give the keratinocytes a selective advantage. The same argument applies to tumour promoters like teleocidin which bind to the phorbol ester receptor (Umezawa *et al.*, 1981; Schmidt *et al.*, 1983).

However, epidermal tumour promoters like anthralin or benzoyl peroxide do not bind to phorbol ester receptors (Delclos *et al.*, 1980) suggesting that their molecular mechanism of action is different from that of TPA. This is further substantiated by the observation that benzoyl peroxide is an efficient promoter in mouse strains where TPA is not (Reiners *et al.*, 1984). Anthralin and benzoyl peroxide do not produce the same morphological effects as TPA in keratinocyte cultures (Parkinson & Emmerson, 1982; Lawrence *et al.*, 1984) but benzoyl peroxide is reported to induce cornification in other systems (Saladino *et al.*, 1985). Nevertheless, as the differential effect of these compounds on normal and transformed keratinocytes *in vitro* has not yet been examined the application of the selection theory (Yuspa *et al.*, 1981; 1982; 1983*b*) to non-phorbol promoters cannot be ruled out.

Diethylstilboestrol is a possible tumour promoter in the human genital tract (Lillehaug & Djurhuus, 1982; Siegfried *et al.*, 1984) and when Stanley *et al.* (1985) studied its effects on cultured cervical keratinocytes and their transformed counterparts, the found it killed tumour cells rather more slowly than normal keratinocytes perhaps indicating that some non phorbol promoters are also selective for transformed cells. However this would be a weak effect compared to that produced by TPA (Stanley *et al.*, 1985).

### 5. Heterogeneity of the reponse of basal keratinocytes to TPA-induced terminal differentiation

It is thought that initiated cells have the phenotype of normal cells (Boutwell, 1974, 1982) or that their transformed character is greatly repressed (see Hennings & Yuspa, 1985, and references therein). This coupled with the observation that initiation is essentially permanent suggests that initated cells are stem cells or committed cells that have irreversibly dedifferentiated into stem cells as a consequence of initiation, because under normal circumstances the stem cells are the only permanent residents of the epidermis (see Potten, 1983, for a review). If initiated cells have a normal phenotype however, the simple version of the selection theory outlined in section 4 is presented with a problem. If TPA were to induce all phenotypically normal keratinocytes to differentiate with equal probability the first promoter treatment would result in the loss through terminal differentiation of many initiated cells before they could be promoted. Whilst this may be the case, it seems more likely that the loss of initiated cells is avoided because they are contained within a keratinocyte sub-population, which also contains the stem cells, and which is refractory or less sensitive to the induction of terminal differentiation by TPA (see Yuspa *et al.*, 1981).

Keratinocyte sub-populations have indeed been reported in which TPA fails to inhibit DNA synthesis and increase transglutaminase activity (Yuspa *et al.*, 1982, 1983*b*) or induce cornified envelope and disulphide bond formation (Reiners & Slaga 1983). Also we have found that when keratinocyte cultures are treated with various doses of TPA, a dose of $10^{-8}$ M is sufficient to eliminate the replicative potential of most of the keratinocytes, but a small sub-population is resistant to doses as high as $10^{-5}$ M (Parkinson *et al.*, 1983; 1984). These $TPA^R$ keratinocytes are the precursor population of the other clonogenic keratinocytes and their differentiated progeny (Parkinson *et al.*, 1983), and show an inverse relationship with the differentiated keratinocytes in a variety of experimental situations (see Parkinson *et al.*, 1984). Yuspa *et al.*, (1982, 1983*b*) have also described a keratinocyte population which fails to respond to TPA-induced terminal differentiation and which regenerates the TPA-responsive population. We have suggested that the $TPA^R$ keratinocytes are a sub-population of clonogenic keratinocytes further removed from terminal differentiation than the rest (Parkinson *et al.*, 1983; 1984) and Yuspa and co-workers have argued along similar lines (Yuspa *et al.*, 1981, 1982, 1983*b*). Two main pieces of evidence support these arguments.

When keratinocytes are separated into different size classes on percoll gradients on the basis of their bouyant density (Sun & Green, 1976) it can be demonstrated that the smallest cells are the ones furthest removed from terminal differentiation (Sun & Green, 1976; Fischer *et al.*, 1982*b*; Schweizer *et al.*, 1984). Exploiting this observation Reiners and Slaga (1983) were able to show that markers of the differentiated state appeared much more quickly in the large mature keratinocytes than in the smaller cells following the separation of mouse keratinocytes from skin treated *in vivo* with promoting doses of TPA.

The second piece of evidence comes from a study of the effect of TPA on basal cells at different points in the keratinocyte lineage *in vivo* (Morris *et al.*, 1985). Mouse dorsal epidermis is composed of numerous epidermal proliferative units (EPU) (see Potten, 1983, for a review) which in turn are composed of a central small group of 3–4 basal cells surrounded by 6 or 7 other basal cells, all of which are covered by a stack of 9 or 10 suprabasal cells at various stages of maturation. (Figure 1). A cell in the central subgroup is a likely candidate for

**Figure 1** The epidermal proliferative unit.

the stem cell of the unit because of its strategic position, slow cell cycle, rapid response to wounding and because it is rarely seen to move suprabasally, in contrast to the peripheral basal cells of the unit (see Potten, 1983). If mouse epidermis is labelled with [$^3$H]TdR over a period of 7 days 95% of the basal cells are labelled (Morris *et al.*, 1985) but if the animals are left for one month the labelling index drops to 2%. Ninety per cent of the latter label-retaining cells (LRCs) as defined by (Mackenzie & Bickenbach, 1982) were located within a nuclear diameter of the central cell position of the EPU (Morris *et al.*, 1985). If the central cell was the stem cell of the EPU it might be expected to have a slower cell cycle time than the peripheral committed cells dividing only once for every two divisions of the latter, and this would partly explain its label-retaining properties. In the experiments of Morris *et al.* (1985) when mouse skin was treated with a promoting dose of TPA soon after the basal cells were labelled, most of the labelled keratinocytes moved suprabasally in line with earlier reports (Argyris, 1980*b*; Reiners & Slaga, 1983) and with the observation that TPA induces a terminal differentiation in the majority of keratinocytes (Steinert & Yuspa, 1978; Yuspa *et al.*, 1980; 1982; Parkinson & Emmerson, 1982; Hawley-Nelson *et al.*, 1982; Mufson *et al.*, 1982; Reiners & Slaga, 1983). If treatment of the animals with TPA was delayed for one month to allow a study of the LRCs then these cells, the majority of which may be stem cells, responded very differently. They rarely moved suprabasally and divided at least once within the 28 h experimental period as evidenced by the appearance of labelled mitoses (Morris *et al.*, 1985). Furthermore, it was possible to demonstrate the persistance of some of the LRCs and their progeny for at least 2 weeks after TPA treatment suggesting that some of the LRCs were responsible for the long term regeneration of the epidermis following TPA-induced cell loss. If the LRCs are stem cells and the initiated keratinocytes are retained within this population then the induction of terminal differentiation in the mature basal keratinocytes coupled with the stimulation of stem cell division (including the initiated ones) would explain how essentially phenotypically normal initiated cells would at first expand into a clone (Yuspa *et al.*, 1981; 1982; 1983*b*). Following this the transformed phenotype would be expressed (Slaga *et al.*, 1980*b*; Furstenberger *et al.*, 1983) and the transformed cells would be then given a selective advantage as outlined in section 4 (see Figure 2). Whether the stem cells (and hence also the initiated cells) are stimulated to divide directly by TPA as suggested by Yuspa *et al.* (1981; 1982; 1983*b*) or indirectly as a consequence of the natural

**Figure 2** The clonal expansion of initiated cells and their progeny.

response of the epidermal tissue to TPA-induced injury (Argyris, 1980*b*) is not entirely certain. The hypothesis of Yuspa *et al.* (1981; 1982; 1983*b*) predicts that because initiated cells are blocked at an early stage of differentiation (see also Yuspa *et al.*, 1985) they should proliferate in reponse to TPA and remain refractory to the stimulus to differentiate. Recent studies have shown however, that whilst the second part of the prediction is fulfilled in cultured initiated cell lines, their proliferative response to TPA is variable (Hennings *et al.*, 1984). Regardless of the precise mechanism, the observation that epidermal proliferation follows TPA treatment of mouse skin *in vivo*, (Raick *et al.*, 1972) is consistent with the selection theory proposed by Yuspa *et al.*

### 6. The selection theory and second stage promoters

At first because stage I of tumour promotion (see section 1) was thought to be irreversible and could be achieved by as little as one treatment with TPA

(Slaga *et al.*, 1980*b*; Furstenberger *et al.*, 1983), it was suggested that TPA caused 'a permanent alteration in the genetic readout of the initiated cell' to complete stage I whereas subsequent TPA treatments were only necessary to make the dormant papilloma cells become visible (Furstenberger *et al.*, 1983). However, the high efficiency by which a single TPA treatment achieves stage I promotion (40% of those initiated – Furstenberger *et al.*, 1981) suggested that a permanent genomic change was unlikely to be involved, and recent data showing stage I of promotion to be reversible, with at most a half-life of 10 weeks (Slaga, 1983; Furstenberger & Marks, personal communication), supports this suggestion.

Yuspa *et al.* (1981) first introduced the concept that second stage promoters might be toxic to the initiated cells and have recently suggested that they might be operative at a later stage because the dose of the second stage promoter would most likely be reduced at later times as a result of the hyperplastic thickening of the epidermis. It was suggested that this, coupled with the increased initiated clone size, would afford protection against second-stage promoter toxicity (Hennings & Yuspa, 1985). These authors further suggested that stage I promotion would be completed spontaneously by limited clonal expansion of the initiated cells so that a second-stage promoter (in this case, mezerein) would be efficient as a complete promoter if its application were delayed. They provided data which supported their hypothesis but their ideas are difficult to reconcile with the recent observation that stage I promotion *is* reversible even when brought about by TPA treatment (Slaga, 1983) whereas the clonal expansion following initiation is not (Loehrke *et al.*, 1983). However, the suggestion that second stage promoters may be more damaging to the initiated cells than TPA is supported by the observations that they inhibit tumour promotion by TPA when applied before it (Sharkey cited by Argyris, 1983) or together with it (Slaga *et al.*, 1980*b*). Furthermore, the $TPA^R$ population in keratinocyte cultures which is similar to the stem cell population *in vivo* as regards its response to TPA (and hence might be representative of the initiated cells – see section 5) is very sensitive to both mezerein and RPA (Parkinson *et al.*, 1984; Stanley *et al.*, 1985). Similarly, preliminary reports suggest that the label-retaining putative stem cells (Mackenzie & Bickenbach, 1982) are not stimulated to divide by either mezerein (McCutcheon *et al.*, 1984) or RPA (McCutcheon *et al.*, 1985) in contrast to their stimulation by TPA (Morris *et al.*, 1985). Dark cells thought by some to primitive keratinocytes (Raick, 1973) are also observed in relatively fewer numbers in mezerein-treated, as opposed to TPA-treated Sencar mouse skin (Klein-Szanto *et al.*, 1980; 1982) and there is evidence that dark cell induction is a good indicator of stage I promoting power in this system (Klein-Szanto *et al.*, 1982; Slaga *et al.*, 1980*a*). The apparent toxic action of second stage promoters may result from their ability to induce terminal differentiation in all the basal cells equally whereas TPA appears to preferentially affect the more mature population (Parkinson *et al.*, 1983; 1984; Morris *et al.*, 1985).

Nevertheless, the observations that RPA at the 10 nmol dose used by Furstenberger and co-workers (Furstenberger *et al.*, 1981, 1983) is not toxic to the initiated cells (Kinzel *et al.*, 1984*a*) and that RPA is 10-fold less potent at inhibiting DNA synthesis in HeLa cells (Kinzel *et al.*, 1984*b*) suggest that second-stage promoters fail as complete promoters because at low doses they do not fulfil a vital function necessary for the completion of stage I promotion, but that when the dose is increased to a level where stage I could be completed they are toxic to the initiated cells and so few papillomas still result (Parkinson *et al.*, 1984). Additional evidence that there are indeed two qualitatively different stages in promotion comes from the observations that the two stages are inhibited by different antagonists (Slaga *et al.*, 1980*a*) and are dependent to different extents on the synthesis of prostaglandins $F_{2\alpha}$ and $E_2$ (Fustenberger & Marks, personal communication). This suggests that the two stages have a different molecular basis. Furthermore, the argument that the second stage promoters fail to promote because they are generally toxic is also not supported by our observation that cell lines from papillomas and carcinomas respond to second stage promoters and TPA in a similar fashion (Parkinson *et al.*, 1984; Stanley *et al.*, 1985). Additionally, transformed cell lines *are* sensitive to the toxic effects of the second stage promoters if the defective response to a differentiation signal is not expressed, as is the case with the non-tumourigenic subclone F of carcinoma line SCC-12 (Parkinson *et al.*, 1984).

Taken together the above data suggest that toxicity may play a part in the failure of RPA and mezerein to promote tumours but that full phenotypic expression of the defective response to inducers of terminal differentiation may also be associated with the early TPA treatments. Whilst a quasi-stable derepression of the initiated phenotype is consistent with this, stage I could also be explained by the selection hypothesis (Yuspa *et al.*, 1981; 1982; 1983*b*; see Figure 3).

The first TPA treatment of mouse skin causes inhibition of DNA synthesis (Raick *et al.*, 1972) and extensive basal cell loss (Argyris, 1980*b*;

**Figure 3** The role of selection in tumour promotion.

Reiners & Slaga, 1983; Morris *et al.*, 1985) probably as a consequence of induced terminal differentiation (see sections 3, 4 and 5). In the hyperplasia that follows, extensive regeneration of the epidermis would occur from the stem cell compartment (including the initiated cells) as discussed earlier (section 5), and could in some way release the initiated cell from the repressive effect of local inhibitory factors (Bell, 1976). In contrast, there is little inhibition of DNA synthesis (and by inference basal cell differentiation) associated with subsequent TPA treatments and the reason for this is unknown. It may be related to an overall change in the epidermal cells themselves brought about by the first TPA treatment causing them to lapse into a ontogentically more primitive state (Marks *et al.*, 1982), where the basal layer is taken over by a chalone insensitive stem cell population (Marks *et al.*, 1978*a, b*) that is stimulated to proliferate by TPA instead of being inhibited (Yuspa *et al.*, 1981; 1982; 1983*b*). Alternatively, there might be a more trivial explanation related to the hyperplastic thickening of the epidermis prior to the second, but not the first TPA treatment. This would have the effect of reducing the magnitude, or rate of delivery of the second dose of TPA, and hence also the inhibition of DNA synthesis and the induction of terminal differentiation in the basal layer, whilst still considerably accelerating differentiation in the superficial layers which do not synthesise DNA. This would have the effect of sustaining a regenerative hyperplasia without prior inhibition of DNA synthesis. This alternative is supported by the observation that hyperplasia caused by the later TPA treatments is still dependent on the synthesis of prostaglandin $E_2$ and this would not be expected if TPA was stimulating DNA synthesis without some prior damage (Marks *et al.*, 1982).

Assuming that the above statements are true then it is clear that the first clonal expansion after TPA would be much larger than the subsequent ones as there would be far more basal cell loss in the former case, removing any lateral restriction to expansion of the initiated clones along the basement membrane. With later treatments the growth of the papilloma would be restricted to an upward direction because of the lateral restriction of the normal basal cells, explaining also the classic pedunculated shape of these lesions. Such an argument is also consistent with the observation that stage I of promotion is much longer lasting than stage II (Slaga *et al.*, 1980*b*; Furstenberger *et al.*, 1983).

To summarise, I am suggesting that in contrast to TPA, second stage promoters are unable to cause the initial selective growth from the stem cell compartment, because they induce terminal differentiation in all the basal cells equally, unlike TPA which selectively induces differentiation in the more mature basal keratinocytes (Parkinson *et al.*, 1983; Parkinson *et al.*, 1984; Morris *et al.*, 1985). We suggest also that the initial clonal expansion leads in some way to the expression of the transformed phenotype. Following this TPA *or* the second stage promoters would give transformed keratinocytes a selective advantage (as in section 4), because when the transformed phenotype is expressed, keratinocytes are equally refractory to TPA- or second stage promoter-induced terminal differentiation (Parkinson *et al.*, 1984).

### 7. The role of selection in tumour promotion

It was recently shown that more than one oncogene must be activated in concert to malignantly transform fibroblasts (Ruley, 1983, Land *et al.*, 1983, Newbold & Overell, 1983). Initiated cells contain at least one of the genomic changes required for keratinocyte malignancy and the selective clonal expansion of these cells into papillomas of $10^5-10^6$ cells (Hennings & Yuspa 1985) would increase the probability that one or more of the initiated cells will acquire the remaining changes required to complete carcinogenesis.

Prior to malignancy papillomas may first acquire the ability to grow autonomously in the differentiating zone of the epidermis (see section 1, Figure 3) and there is some evidence that complementation of an activated *ras*$^H$ gene by immortalisation and/or aneuploidy (Weissman & Aaronson, 1983) allows keratincoytes to grow under conditions where *ras*$^H$ activation alone cannot (Yuspa *et al.*, 1983*a*, 1985). Cell lines cultured from papillomas also display both *in vitro* immortality (Pera & Gorman, 1984) and an activated *ras*$^H$ gene (M.F. Pera & A. Balmain – personal communication). An increased ability to evade senescence when cultured in the presence of low levels of serum growth factors is a property associated with fibroblast immortality and in this cell type a combination of immortalisation and an activated *ras*$^H$ gene is sufficient to give malignancy (R.F. Newbold – personal communications). One could speculate that a reduced requirement for certain growth factors might also allow keratinocytes initiated by *ras*$^H$ activation to grow autonomously in zones of the epidermis where previously they could not (see Yuspa *et al.*, 1985). Whether the irreversible change from a promoter-dependent to a promoter-independent lesion is spontaneous or whether it involves TPA-induced chromosome changes (Kinsella & Radman, 1978; Emeritt & Cerutti, 1981; Parry *et al.*, 1981) or gene amplications (Varshavsky, 1981) as a possible consequence of the ability of TPA to generate free radicals (Troll *et al.*, 1982) awaits the design of suitable *in vivo* experiments as far as epidermis is concerned. It is interesting, however, that TPA (Colburn *et al.*, 1978 and other free radical generating compounds (Gindhart *et al.*, 1984) can induce certain epidermal cell lines to become irreversibly anchorage independent, and this might be relevent to the ability of keratinocytes to grow autonomously even when detached from the basement membrane. Furthermore, if involved at all, free radicals are thought to be involved in stage II of promotion (Schwarz *et al.*, 1984) and the effect of TPA on anchorage independence is blocked by both radical scavengers (Nakamura *et al.*, 1985) and retinoids (Colburn, 1979) the last of which specifically inhibits stage II promotion (Slaga *et al.*, 1980*a*).

Nevertheless since cell lines derived from papillomas (unlike those from carcinomas) are still not tumourigenic (Pera & Gorman, 1984) it is clear that a third irreversible event is required to produce malignancy in keratinocytes in contrast to the situation with fibroblasts (Newbold & Overell, 1983). This third event may only be necessary in epithelium because of the constraints placed upon the tumour cells by the close proximity of normal cells in this tissue as fully discussed elsewhere by Balmain (1985), and in mouse skin carcinogenesis the evidence uniformily suggests that malignant conversion proceeds independently of TPA once the maximum papilloma yield has been obtained (Burns *et al.*, 1978; Verma & Boutwell, 1980; Hennings *et al.*, 1983).

In this review I have attempted to present evidence supporting the hypothesis that clonal selection of the initiated cells (Yuspa *et al.*, 1981) is important for tumour promotion by TPA and wounding. It should be stressed however, that these ideas may not necessarily apply to promoters outside the diterpene series (Delclos *et al.*, 1980; Reiners *et al.*, 1984) or indeed to tissues other than those capable of cornification. A better assessment of the arguments put forward in this article will depend of course upon future developments in oncogene and growth factor research, but will also require an improvement in our knowledge of the keratinocyte cell lineage and in particular its response to injury.

I would like to thank; Professor D.G. Harnden, Dr W.J. Hume, Dr A.R. Kinsella and Dr C.S. Potten for reviewing the manuscript, Mrs I. Nicholls for artwork and photography, Drs A. Balmain, G. Furstenberger, C.C. Harris, F. Marks, R.F. Newbold, M.F. Pera and S.H. Yuspa for allowing me to quote their unpublished data and Miss M.A. Spilling for typing the manuscript.

I am also indebted to Dr C.S. Potten and Churchill Livingstone Publications for the reproduction of the diagram of the epidermal proliferative unit, and to the Cancer Research Campaign for continued financial support.

### References

ARGYRIS, T.S. (1980*a*). Tumour promotion by abrasion induced epidermal hyperplasia in the skin of mice. *J. Invest. Dermatol.*, **75**, 360.

ARGYRIS, T.S. (1980*b*). Epidermal growth following a single application of 12-0-tetradecanoyl-phorbol-13-acetate in mice. *Am. J. Pathol.*, **98**, 639.

ARGYRIS, T.S. (1983). Nature of the epidermal hyperplasia produced by mezerein, a weak tumour promoter, in initiated skin of mice. *Cancer Res.*, **43**, 1768.

ASSOIAN, R.K., KOMORIYA, A., MEYERS, C.A., MILLER, D.M. & SPORN, M.B. (1983). Transforming growth factor in human platelets. *J. Biol. Chem.*, **258**, 7155.

BALMAIN, A. (1985). Transforming ras oncogenes and multistage carcinogenesis. *Br. J. Cancer*, **51**, 1.

BALMAIN, A. & PRAGNELL, I.B. (1983). Mouse skin carcinomas induced *in vivo* by chemical carcinogens have a transforming Harvey-ras oncogene. *Nature*, **303**, 72.

BALMAIN, A., RAMSDEN, M., BOWDEN, G.T. & SMITH, J. (1984). Activation of the mouse cellular Harvey-ras gene in chemically induced benign skin papillomas. *Nature*, **307**, 658.

BELL, G.I. (1976). Models of carcinogenesis as an escape from mitotic inhibitors, *Science*, **192**, 569.

BELMAN, S. & TROLL, W. (1972). The inhibition of croton-oil promoted mouse skin tumourigenesis by steroid hormones. *Cancer Res.*, **32**, 450.

BERENBLUM, I. & SHUBIK, P. (1947). The role of croton oil applications associated with a single painting of a carcinogen, in tumour induction of the mouse's skin. *Br. J. Cancer*, **1**, 379.

BERTSCH, S. & MARKS, F. (1974). Lack of an effect of tumour-promoting phorbol esters and epidermal $G_1$ chalone on DNA synthesis in the epidermis of newborn mice. *Cancer Res.*, **34**, 3283.

BOUTWELL, R.K. (1964). Some biological aspects of skin carcinogenesis. *Prog. Exp. Tumour, Res.*, **4**, 207.

BOUTWELL, R.K. (1974). The function and mechanism of promoters of carcinogenesis. *CRC Crit. Rev. Toxicol.*, **2**, 419.

BOUTWELL, R.K. VERMA, A.K. ASHENDEL, C.L. & ASTRUP, E. (1982). Mouse skin: A useful model system for studying the mechanism of chemical carcinogenesis. In *Carcinogenesis*, Vol. 7, Hecker E. *et al.* (eds.) p. 1. Raven Press: New York.

BURNS, F.J., VANDERLANN, M., SNYDER, E., ALBERT, R.E. (1978). Induction and progression kinetics of mouse skin papillomas. In *Mechanisms of Tumour Promotion and Cocarcinogenesis*, Vol. 2, Slaga, T.J. *et al.* (eds.) p. 91. Raven Press: New York.

CASTAGNA, M., TAKAI, Y., KAIBUCHI, K., SANO, K., KIKKAWA, U., NISHIZUKA, Y. (1982). Direct activation of calcium-activated, phospholipid dependent protein kinase by tumor promoting phorbol esters. *J. Biol. Chem.*, **257**, 7847.

CHILDS, B.J., PROPER, J.A., TUCKER, R.F. & MOSES, H.L. (1982). Serum contains a platelet-derived transforming growth factor. *Proc. Natl. Acad. Sci. USA*, **79**, 5312.

CLARK-LEWIS, I. & MURRAY, A.W. (1978). Tumor promotion and the induction of epidermal orithine decarboxylase activity in mechanically stimulated mouse skin. *Cancer Res.*, **38**, 494.

COLBURN, N.H. (1979). The use of tumor promoter – responsive epidermal cell lines to study preneoplastic progression. In *Neoplastic Transformation in Differentiated Epithelial Cell Systems* in vivo, Franks, L.M. & Wigley, C.B. (eds) p. 113, Academic Press: New York.

COLBURN, N.H., FORMER, B.F., NELSON, K.A. & YUSPA, S.H. (1978). Tumor promoter induces anchorage independence irreversibly. *Nature*, **281**, 589.

DELCLOS, K.B., NAGLE, D.S. & BLUMBERG, P.M. (1980). Specific binding of phorbol ester tumor promoters to mouse skin. *Cell*, **19**, 1025.

DI GIOVANNI, J., PRICHETT, W.P., DECINA, P.C. & DIAMOND, L. (1984). DBA/2 mice are as sensitive as SENCAR mice to skin tumor promotion by 12-0-tetra decanoylphorbol-13-acetate. *Carcinogenesis*, **5**, 1493.

DUESBERG, P.H. (1983). Retroviral transforming genes in normal cells? *Nature*, **304**, 219.

EBELING, J.G., VANDERBARK, G.R., KUHN, L.J., GANONG, B.R., BELL, R.M. & NIEDEL, J.E. (1985). Diacylglycerols mimic phorbol diester induction of leukemic cell differentiation. *Proc. Natl Acad. Sci.*, (USA) **82**, 815.

EMERITT, I. & CERUTTI, P. (1981). Tumor promoter phorbol, 12-myristate, 13-acetate induces chromosome damage by indirect action. *Nature*, **293**, 144.

FISCHER, S.M., MILLS, G.D. & SLAGA, T.J. (1982*a*). Inhibition of mouse skin tumor promotion by several inhibitors of arachidomic acid metabolism. *Carcinogenesis*, **3**, 1243.

FISCHER, S.M., NELSON, K.D.G., REINERS, J.J., VIAJE, A., PELLING, J.C. & SLAGA, T.J. (1982*b*). Separation of epidermal cells by density centrifugation: A new technique for studies on normal and pathological differentiation. *J. Cutaneous Path.*, **9**, 43.

FUERSTEIN, N. & COOPER, H.L. (1984). Rapid phosphorylation – dephosphorylation of specific proteins induced by phorbol ester in HL-60 cells. Further characterization of the phosphorylation of 17-kilodalton and 27-kilodalton proteins in myeloid leukemic cells and human monocytes. *J. Biol. Chem.*, **259**, 2782.

FURSTENBERGER, G., BERRY, D.L., SORG, B. & MARKS, F. (1981). Skin tumor promotion by phorbol esters is a two-stage process. *Proc. Natl Acad. Sci.* (USA), **78**, 7722.

FURSTENBERGER, G. & MARKS, F. (1978). Indomethacin inhibition of cell proliferation induced by the phorbol ester TPA is reversed by prostaglandin $E_2$ in mouse epidermis *in vivo*. *Biochem. Biophys. Res. Comm.*, **92**, 749.

FURSTENBERGER, G., SCHWEIZER, J. & MARKS, F. (1985). Development of phorbol ester responsiveness in neonatal mouse epidermis: Correlation between hyperplastic response and sensitivity to first stage tumor promotion. *Carcinogenesis*, **6**, 289.

FURSTENBERGER, G., SORG, B. & MARKS, F. (1983). Tumor promotion by phorbol esters in skin: Evidence for a memory effect. *Science*, **220**, 89.

FREI, J.V. & STEPHENS, P. (1968). The correlation of promotion of tumour growth and induction of hyperplasia in epidermal two-stage carcinogenesis. *Br. J. Cancer*, **22**, 83.

GINDHART, T.G., SRINIVAS, L. & COLBURN, N.H. (1984). Benzoyl peroxide induced promotion of transformation in JB6 mouse epidermal cells, *Carcinogenesis*, **6**, 309.

GOERTTLER, K., LOEHRKE, H., HESSE, B. & SCHWEIZER, J. (1984). Skin tumor formation in the European hamster (Cricetus Cricetus, L.) after topical initiation with, 7,12 dimethylbenz(a) anthracene (DBMA) and promotion with 12.0-tetradecanoyl phorbol-13-acetate (TPA). *Carcinogenesis*, **5**, 521.

HAWLEY-NELSON, P., STANLEY, J.R., SCHMIDT, J., GULLINO, M. & YUSPA, S.H. (1982). The tumor promoter, 12-0-tetradecanoylphorbol-13-acetate accelerates keratinocyte differentiation and stimulates growth of an unidentified cell type in cultured human epidermis. *Exp. Cell Res.*, **137**, 115.

HENNINGS, H., BEN, T., YUSPA, S.H. (1984). Treatment of epidermal cell lines with 12-0-tetradecanoylphorbol-13-acetate (TPA): lack of differentiative response and variable proliferative response. *Proc. Amer. Assoc. Cancer Res.*, **25**, 146 (*abstract*).

HENNINGS, H. & BOUTWELL, R.K. (1967). On the mechanism of inhibition of benign and malignant skin tumour formation by actinomycin D. *Life Sci.*, **6**, 173.

HENNINGS, H. & BOUTWELL, R.K. (1970). Studies on the mechanism of skin tumor promotion. *Cancer Res.*, **12**, 312.

HENNINGS, M. MICHAEL, D., CHENG, C., STEINERT, P. HOLBROOK, K. & YUSPA, S.H. (1980). Calcium regulation of growth and differentiation of mouse epidermal cells in culture. *Cell*, **19**, 245.

HENNINGS, H., SHORES, R., WENK, M.L., SPANGLER, E.F., TARONE, R. & YUSPA, S.H. (1983). Malignant conversion of mouse skin tumours is increased by tumour initiators and unaffected by tumour promoters. *Nature*, **304**, 67.

HENNINGS, H. & YUSPA, S.H. (1985). Two stage tumor promotion in mouse skin: An alternative interpretation. *J. Natl Cancer Inst.*, **74**, 735.

IANNACONE, P.M., GARDNER, R.L. & HARRIS, H. (1978). The cellular origin of chemically induced tumors. *J. Cell Sci.*, **29**, 249.

KINSELLA, A.R. & RADMAN, M. (1978). Tumor promoter induces sister chromatial exchanges: Relevence to mechanisms of carcinogenesis. *Proc. Natl Acad. Sci.* (USA), **75**, 6149.

KINZEL, V., LOEHRKE, H., GOERTTLER, K., FURSTENBERGER, G. & MARKS, F. (1984*a*). Suppression of the first stage of phorbol 12-tetradecanoate-13-acetate-effected tumor promotion in mouse skin by non-toxic inhibition of DNA synthesis. *Proc. Natl Acad. Sci.* (USA), **81**, 5858.

KINZEL, V., RICHARDS, J., MARKS, F. & FURSTENBERGER, G. (1984*b*). Radiomimetic activity of phorbol esters exerted in HeLa cells in comparison with their tumor-promoting capacity. *Cancer Res.*, **44**, 139.

KLEIN-SZANTO, A.J.P., MAJOR, S.K. & SLAGA, T.J. (1980). Induction of dark keratinocytes by 12-0-tetradecanoyl phorbol-13-acetate and mezerein as an indicator of tumor promoting efficiency. *Carcinogenesis*, **1**, 399.

KLEIN-SZANTO, A.J.P., MAJOR, S.K. & SLAGA, T.J. (1982). Quantitative evaluation of dark keratinocytes induced by several promoting and hyperplasiogenic agents: Their use as an early morphological indicator of tumor-promoting action. In *Carcinogenesis*, Vol. 7, Hecker E. *et al.* (eds) p. 305. Raven Press: New York.

KRIEG, L., KUHLMANN, I. & MARKS, F. (1974). Effect of tumour-promoting phorbol esters and of acetic acid on mechanisms controlling DNA synthesis and mitosis (chalones) and on biosynthesis of histidine-rich protein in mouse epidermis. *Cancer Res.*, **34**, 3135.

LAND, H., PARADA, L.F. & WEINBERG, R.A. (1983). Tumorigenic conversion of primary embryo fibroblasts requires at least two co-operating oncogenes. *Nature*, **304**, 596.

LAWRENCE, N.J., PARKINSON, E.K. & EMMERSON, A. (1984). Benzoyl peroxide interferes with metabolic cooperation between cultured human keratinocytes. *Carcinogenesis*, **5**, 419.

LECHNER, J.F., McCLENDON, I.A., LAVECK, M.A., SHAMSUDDIN, A.M. & HARRIS, C.C. (1983). Differential control by platelet factors of squamous differentiation in normal and malignant human bronchial epithelial cells. *Cancer Res.*, **43**, 5915.

LILLEHAUG, J.R. & DJURHUUS, R. (1982). Effect of diethylstilbrestrol on the transformable mouse embryo fibroblast C3H/10T½2C18 cells. Tumour promotion, cell growth, DNA synthesis and ornithine decarboxylase. *Carcinogenesis*, **3**, 797.

LOEHRKE, H., SCHWEIZER, J., DEDENDER, E., HESSE, B., ROSENKRANZ, G. & GOERTTLER, K. (1983). On the persistance of tumor initiation in two stage carcinogenesis on mouse skin. *Carcinogenesis*, **4**, 771.

MACKENZIE, I.C. & BICKENBACH, J.R. (1982). Patterns of epidermal cell proliferation. In *Carcinogenesis*, Vol. 7, Hecker E. *et al.* (eds) p. 331. Raven Press: New York.

MARKS, F., BERTSCH, S. & FURSTENBERGER, G. (1979). Ornithine decarboxylase activity, cell proliferation and tumor promotion in mouse epidermis *in vivo*. *Cancer Res.*, **39**, 4183.

MARKS, F., BERTSCH, S., GRIMM, W. & SCHWEIZER, J. (1978*a*). Hyperplastic transformation and tumor promotion in mouse epidermis: Possible consequences of disturbances of endogenous mechanisms controlling proliferation and differentiation. In *Carcinogenesis*, Vol. 2, Slaga, T.J. *et al.* (eds) p. 97. Raven Press: New York.

MARKS, F., BERTSCH, S. & SCHWEIZER, J. (1978*b*). Homeostatic regulation of epidermal cell proliferation. *Bull. Cancer* (Paris), **65**, 207.

MARKS, F., BERRY, D.L., BERTSCH, S., FURSTENBERGER, G. & RICHTER, H. (1982). On the relationship between epidermal hyperproliferation and skin tumour promotion. In *Carcinogenesis*, Vol. 7, Hecker E. *et al.* (eds) p. 331. Raven Press: New York.

MARKS, F., FURSTENBERGER, G. & MARKS, F. (1981). Prostaglandin, E-mediated mitogenic stimulation of mouse epidermis *in vivo* by divalent cation inophore A23187 and by tumor promoter TPA. *Cancer Res.*, **41**, 696.

McCUTCHEON, J.A., BICKENBACH, J.R. & MACKENZIE, I.C. (1984). Effects of hyperplasiogens on epithelial label-retaining cells. *J. Dental Res.*, **63**, 261 (*abstract*).

McCUTCHEON, J.A., BICKENBACH, J.C. & MACKENZIE, I.C. (1985). Effect on label retaining cells of tumor promoters and differing levels of hyperlasia. *J. Dental Res.*, **64**, 298. (*abstract*).

MORRIS, R.J. & ARGYRIS, T.S. (1983). Epidermal cell cycle and transit times during hyperplastic growth induced by abrasion or treatment with 12-0-tetradecanoylphorbol-13-acetate. *Cancer Res.*, **43**, 4935.

MORRIS, R.J., FISCHER, S.M. & SLAGA, T.J. (1985). Evidence that the centrally and peripherally located cells in the murine epidermal proliferative unit are two distinct cell populations. *J. Invest. Dermatol.*, **84**, 277.

MUFSON, R.A., FISCHER, S.M., VERMA, A.K., GLEASON, G.L., SLAGA, T.A. & BOUTWELL, R.K. (1979). Effects of 12-0-tetradecanoylphorbol-13-acetate and mezerein on epidermal ornithine decarboxylase activity, isoproterenol stimulated levels of cyclic adenosine 3′5′ monophosphate and induction of mouse skin tumours. *in vivo*. *Cancer Res.*, **39**, 4791.

MUFSON, R.A., STEINBERG, M.L. & DEFENDI, V. (1982). Effects of 12-0-tetradecanoylphorbol-13-acetate on the differentiation of Simian Virus 40 infected human keratinocytes. *Cancer Res.*, **42**, 4600.

MURRAY, A.W. (1978). Acetic acid pretreatment of initiated epidermis inhibits tumor promotion by a phorbol ester. *Experientia*, **34**, 1507.

NAKAMURA, Y., COLBURN, N.H. & GINDHART, T.D. (1985). Role of reactive oxygen in tumor promotion: Implication of superoxide anion in promotion of neoplastic transformation in JB-6 cells by TPA. *Carcinogenesis*, **6**, 229.

NISHIZUKA, Y. (1984). The role of protein kinase C in cell surface signal transduction and tumour promotion. *Nature*, **308**, 693.

NEWBOLD, R.F. & OVERELL, R.W. (1983). Fibroblast immortality is a prerequisite for transformation by EJ c-Ha-ras oncogene. *Nature*, **304**, 648.

PARKINSON, E.K. & EMMERSON, A. (1982). The effects of tumor promoters on the multiplication and morphology of cultured human epidermal keratinocytes. *Carcinogenesis*, **3**, 525.

PARKINSON, E.K., GRABHAM, P. & EMMERSON, A. (1983). A subpopulation of cultured human keratinocytes which is resistant to the induction of terminal differentiation – related changes by phorbol, 12-myristate, 13-acetate: Evidence for an increase in the resistant population following transformation. *Carcinogenesis*, **4**, 857.

PARKINSON, E.K., PERA, M.F., EMMERSON, A. & GORMAN, P.A. (1984). Differential effects of complete and second-stage tumour promoters in normal but not transformed human and mouse keratinocytes. *Carcinogenesis*, **5**, 1071.

PARKINSON, E.K. & EMMERSON, A. (1984). Non promoting hyperplasiogenic agents do not mimic the effects of phorbol, 12-myristate, 13-acetate on terminal differentiation of normal and transformed human keratinocytes. *Carcinogenesis*, **5**, 687.

PARRY, J.M., PARRY, E.M. & BARRETT, J.C. (1981). Tumour promoters induce mitotic aneuploidy in yeast. *Nature*, **294**, 263.

PERA, M.F. & GORMAN, P.A. (1984). *In vitro* analysis of multistage epidermal carcinogenesis: Development of indefinite renewal capacity and reduced growth factor requirements in colony forming keratinocytes precedes malignant transformation. *Carcinogenesis*, **5**, 671.

POTTEN, C.S. (1983). Stem cells in epidermis from the back of the mouse. In *Stem cells, Their Identification and Characterization*, Potten, C.S. (ed.) p. 200. Churchill Livingstone: New York.

RAICK, A.N. (1973). Late ultrastructural changes induced by 12-0-tetradecanoyl phorbol-13-acetate in mouse epidermis and their reversal. *Cancer Res.*, **33**, 1096.

RAICK, A.N. (1974). Cell proliferation and promoting action in skin carcinogenesis. *Cancer Res.*, **34**, 920.

RAICK, A.N. & BURDZY, (1973). Ultrastructural and biochemical changes induced in mouse epidermis by a hyperplastic agent, ethylphenylpropiolate. *Cancer Res.*, **33**, 2221.

RAICK, A.N., THUMM, K. & CHIVERS, B.R. (1972). Early effects of 12-0-tetradecanoylphorbol-13-acetate on the incorporation of tritiated precursor into DNA and the thickness of the interfollicular epidermis and their relation to tumour promotion in mouse skin. *Cancer Res.*, **32**, 1562.

REDDY, A.L. & FIALKOW, P.J. (1983). Papillomas induced by initiation – promotion differ from those induced by carcinogen alone. *Nature*, **304**, 69.

REINERS, J.J., NESNOW, S. & SLAGA, T.J. (1984). Murine susceptibility to two-stage skin carcinogenesis is influenced by the agent used for promotion. *Carcinogenesis*, **5**, 301.

REINERS, J.J. & SLAGA, T.J. (1983). Effects of tumor promoters on the rate and commitment to terminal differentiation of subpopulations of murine keratinocytes. *Cell*, **32**, 247.

RHEINWALD, J.G. & BECKETT, M.A. (1980). Defective terminal differentiation in culture as consistent and selectable character of malignant human keratinocytes. *Cell*, **22**, 629.

RULEY, E.H. (1983). Adenovirus early region 1A enables viral and cellular transforming genes to transform primary cells in culture. *Nature*, **304**, 602.

SALADINO, A.J. WILLEY, J.C. LECHNER, J.F. GRAFTSTROM, R.C. LAVECK, M. & HARRIS, C.C. (1985). Effects of formaldehyde, acetaldehyde, benzoyl peroxide and hydrogen peroxide on cultured normal human bronchial epithelial cells. *Cancer Res.*, **45**, (In Press).

SCHMIDT, R., ADOLF, W., MARSTON, A., ROESER, H., SORG, B., FUJIKI, H., SUGIMURA, T., MOORE, R.E. & HECKER, E. (1983). Inhibition of specific binding of ($^3$H) phorbol-12, 13-dipropionate to an epidermal fraction by certain irritants and irritant promoters of mouse skin. *Carcinogenesis*, **4**, 77.

SCHWARZ, M., PERES, G., KUNZ, W., FURSTENBERGER, G., KITTSTEIN, W. & MARKS, F. (1984). One the role of superoxide anion radicals in skin tumour promotion. *Carcinogenesis*, **5**, 1163.

SCHWARZ, J.A., VIAJE, A., SLAGA, T.J., YUSPA, S.H., HENNINGS, H. & LICHTI, U. (1977). Fluocinolone acetonide: A potent inhibitor of skin tumor promotion and epidermal DNA synthesis. *Chem Biol. Interact*, **17**, 331.

SCHWEIZER, J. KINJO, M., FURSTENBERGER, G. & WINTER, H. (1984). Sequential expression of mRNA-encoded keratin sets in neonatal mouse epidermis: Basal cells with properties of terminally differentiating cells. *Cell*, **37**, 159.

SCHWEIZER, J., LOEHRKE, H. & GOERTTLER, K. (1982). DMBA-TPA mediated skin tumor initiation and promotion in male Sprague–Dawley rats. *Carcinogenesis*, **3**, 785.

SCRIBNER, J.D. & SLAGA, T.J. (1973). Multiple effects of dexamethasone on protein synthesis and hyperlasia caused by a tumor promoter. *Cancer Res.*, **33**, 542.

SCRIBNER, J.D. & SUSS, R. (1978). Tumour initiation and promotion. In *International Review of Experimental Pathology*, Vol. 18, Richter & Epsteins (eds) p. 137. Academic Press: New York.

SIEGFRIED, J.M., NELSON, K.G., MARTIN, J.L. & KAUFMANN, D.G. (1984). Promotional effect of diethylstilboestrol on human endometrial stromal cells pretreated with a direct acting carcinogen. *Carcinogenesis*, **5**, 741.

SISSKIN, E.E. & BARRETT, J.C. (1981). Hyperplasia of Syrian hamster epidermis induced by single but not multiple treatment with 12-0-tetradecanoylphorbol-13-acetate. *Cancer Res.*, **41**, 346.

SISSKIN, E.E., GRAY, T. & BARRETT, J.C. (1982). Correlation between sensitivity to tumor promotion and sustained epidermal hyperplasia of mice and rats treated with 12-0-tetradecanoylphorbol-13-acetate, *Carcinogenesis*, **3**, 403.

SPANDIDOS, D. & WILKIE, N. (1984). *In vitro* malignant transformation of early passage rodent cells by a single mutated human oncogene. *Nature*, **310**, 469.

SLAGA, T.J. (1983). Overview of tumor promotion in animals. *Environ. Health Perspect.*, **50**, 3.

SLAGA, T.J., BOWDEN, G.T. & BOUTWELL, R.K. (1975). Acetic acid, a potent stimulator of mouse epidermal macromolecular synthesis and hyperplasia but with weak tumor-promoting ability. *J. Natl Cancer Inst.*, **55**, 983.

SLAGA, T.J., FISCHER, S.M., NELSON, K. & GLEASON, G.L. (1980*b*). Studies on the mechanism of skin tumor promotion: Evidence for several stages in promotion. *Proc. Natl Acad. Sci.* (USA), **77**, 3659.

SLAGA, T.J., KLEIN-SZANTO, A.J.P., FISCHER, S.M., WEEKS, C.E., NELSON, K. & MAJOR, S. (1980*a*). Studies on mechanism of action of anti-tumor promoting agents: Their specificity in two stage promotion. *Proc. Natl Acad. Sci.* (USA), **77**, 2251.

SLAGA, T.J. & SCRIBNER, (1973). Inhibition of tumor initiation and promotion by anti-inflammatory agents. *J. Natl Cancer Inst.*, **51**, 1723.

SPORN, M.B., ROBERTS, A.B., SCHULL, J.H., SMITH, J.M., WARD, J.M. & SODEK, J. (1983). Polypeptide transforming growth factors isolated from bovine sources and used for wound healing *in vivo*. *Science*, **219**, 1329.

STANLEY, M.A., CROWCROFT, N.S., QUIGLEY, J.P. & PARKINSON, E.K. (1985). Responses of human cervical keratinocytes *in vitro* to tumour promoters and diethylstilboestrol. *Carcinogenesis*, **6**, (In Press).

STEINERT, P. & YUSPA, S.H. (1978). Biochemical evidence for keratinisation by mouse epidermal cells in culture. *Science*, **200**, 1491.

STENBACK, F., GARCIA, H. & SHUBIK, P. (1974). Present status of the concept of promoting action and co-carcinogenesis in skin. In *The Physiopathology of Cancer*, Vol. I, Biology and Biochemistry, Shubik, P. (ed) p. 155. Karger: Basel.

SUN, T.T. & GREEN, H. (1976). Differentiation of the epidermal keratinocyte in cell culture: Formation of the cornified envelope, *Cell*, **9**, 511.

TROLL, W., WITZ, G., GOLDSTEIN, B., STONE, D., SUGIMURA, T. (1982). The role of free radicals in tumor promotion and carcinogenesis, In *Carcinogenesis*, Vol. 7, Hecker, E. *et al.* (eds) p. 593. Raven Press: New York.

TROSKO, J.E., CHANG, C.C., YOTTI, L.P. & CHU, E.H.Y. (1977). Effect of phorbol myristate acetate on the recovery of spontaneous and ultra violet light-induced 6-Thioguanine and Ouabain-resistant Chinese Hamster cells. *Cancer Res.*, **37**, 188.

UMEZAWA, K., WEINSTEIN, I.B., HOROWITZ, A., FUJIKI, H., MATSUSHIMA, T. & SUGIMURA, T. (1981). Similarity of teleocidin B and phorbol ester tumour promoters on membrane receptors. *Nature*, **290**, 411.

VAN DUUREN, B.L., SIVAK, A., KATZ, C., SEIDMAN, I. & MELCHIONNE, S. (1975). The effect of ageing and interval between primary and secondary treatment in two-stage carcinogenesis in mouse skin. *Cancer Res.*, **35**, 502.

VAN DUUREN, B.L., SIVAK, A., LANGSETH, L. (1967). The tumour-promoting activity of tobacco leaf extract and whole cigarette tar. *Br. J. Cancer*, **21**, 502.

VARSHAVSKY, A. (1981). Phorbol ester dramatically increases incidence of methotrexate-resistant mouse cells: Possible mechanisms and relevence to tumor promotion. *Cell*, **25**, 561.

VERMA, A.K., ASHENDEL, C.L. & BOUTWELL, R.K. (1977). Inhibition by prostaglandin synthesis inhibitors of the induction of epidermal ornithine decarboxylase activity, the accumulation of prostaglandins, and tumor promotion caused by TPA. *Cancer Res.*, **40**, 308.

VERMA, AK. & BOUTWELL, R.K. (1980). Effect of dose and duration of treatment with the tumor promoting agent, 12-0-tetradecanoylphorbol-13-acetate on mouse skin carcinogenesis. *Carcinogenesis*, **1**, 271.

WEISSMAN, B.E. & AARONSON, S.A. (1983). Balb and Kirsten murine sarcoma viruses alter growth and differentiation of epidermal growth factor (EGF)-dependent Balb/c mouse epidermal keratinocyte lines. *Cell*, **32**, 599.

WILLEY, J.C., MOSER, C.E., LECHNER, J.F. & HARRIS, C.C. (1984). Differential effects of 12-0-tetradecanoyl-phorbol-13-acetate on cultured normal and neoplastic human bronchial epithelial cells. *Cancer Res.*, **44**, 5124.

YUSPA, S.H., BEN, T., HENNINGS, H. & LICHTI, U. (1980). Phorbol ester tumor promoters induce epidermal transglutaminase activity. *Biochem. Biophys. Res. Commun.*, **97**, 700.

YUSPA, S.H., BEN, T., HENNINGS, H. & LICHTI, U. (1982). Divergent responses in epidermal basal cells exposed to the tumor promoter 12-0-tetradecanoylphorbol-13-acetate. *Cancer Res.*, **42**, 2344.

YUSPA, S.H., HENNINGS, H. & LICHTI, U. (1981). Initiator and promoter induced specific changes in epidermal function and biological potential. *J. Supramol. Struct. Cell Biochem.*, **17**, 245.

YUSPA, S.H., KILKENNY, A.E., STANLEY, J., LICHTI, U. (1985). Keratinocytes blocked in phorbol ester-reponsive early stage of terminal differentiation by sarcoma viruses. *Nature*, **314**, 459.

YUSPA, S.H., KULESZ-MARTIN, M., BEN, T. & HENNINGS, H. (1983*b*). Transformation of epidermal cells in culture. *J. Invest. Dermatol.*, **81**, 162s.

YUSPA, S.H. & MORGAN, D. (1981). Mouse skin cells resistant to terminal differentiation associated with initiation of carcinogenesis. *Nature*, **293**, 72.

YUSPA, S.H., VASS, W. & SCOLNICK, E. (1983*a*). Altered growth and differentiation of cultured mouse epidermal cells infected with oncogenic retrovirus: Contrasting effects of viruses and chemicals. *Cancer Res.*, **43**, 6021.